# Dynamic regulation of histone modifications and long-range chromosomal interactions during postmitotic transcriptional reactivation

**DOI:** 10.1101/gad.335794.119

**Published:** 2020-07-01

**Authors:** Hyeseon Kang, Maxim N. Shokhirev, Zhichao Xu, Sahaana Chandran, Jesse R. Dixon, Martin W. Hetzer

**Affiliations:** 1 Molecular and Cell Biology Laboratory, Salk Institute for Biological Studies, La Jolla, California 92037, USA;; 2 The Razavi Newman Integrative Genomics and Bioinformatics Core (IGC), Salk Institute for Biological Studies, 92037 La Jolla, California, USA;; 3 Peptide Biology Laboratory, Salk Institute for Biological Studies, La Jolla, California 92037, USA

**Keywords:** chromatin, mitosis, transcription

## Abstract

During mitosis, transcription of genomic DNA is dramatically reduced, before it is reactivated during nuclear reformation in anaphase/telophase. Many aspects of the underlying principles that mediate transcriptional memory and reactivation in the daughter cells remain unclear. Here, we used ChIP-seq on synchronized cells at different stages after mitosis to generate genome-wide maps of histone modifications. Combined with EU-RNA-seq and Hi-C analyses, we found that during prometaphase, promoters, enhancers, and insulators retain H3K4me3 and H3K4me1, while losing H3K27ac. Enhancers globally retaining mitotic H3K4me1 or locally retaining mitotic H3K27ac are associated with cell type-specific genes and their transcription factors for rapid transcriptional activation. As cells exit mitosis, promoters regain H3K27ac, which correlates with transcriptional reactivation. Insulators also gain H3K27ac and CCCTC-binding factor (CTCF) in anaphase/telophase. This increase of H3K27ac in anaphase/telophase is required for posttranscriptional activation and may play a role in the establishment of topologically associating domains (TADs). Together, our results suggest that the genome is reorganized in a sequential order, in which histone methylations occur first in prometaphase, histone acetylation, and CTCF in anaphase/telophase, transcription in cytokinesis, and long-range chromatin interactions in early G1. We thus provide insights into the histone modification landscape that allows faithful reestablishment of the transcriptional program and TADs during cell division.

## Abstract (precis)

In this study, Kang et al. sought to understand the underlying principles that mediate transcriptional memory and reactivation in the daughter cells after mitosis. They used ChIP-seq on synchronized cells at different stages after mitosis to generate genome-wide maps of histone modifications, and combined with EU-RNA-seq and Hi-C analyses, they found that during prometaphase promoters, enhancers, and insulators retain H3K4me3 and H3K4me1, while losing H3K27ac, thus providing new insights into the histone modification landscape during cell division.

Mitosis marks a dramatic transition during which cells move from a transcriptionally active to a largely repressed state in which most genes are transcriptionally inactive (Prescott and Bender 1962). This drastic reduction in transcriptional activity is associated with chromosome condensation (Vagnarelli 2013), loss of long-range DNA interactions (Naumova et al. 2013; Dileep et al. 2015; Gibcus et al. 2018), and exclusion of RNA polymerase II (Pol II) and most transcription factors (TFs) from chromatin (Gottesfeld and Forbes 1997). As cells exit mitosis and reform the nuclear envelope (NE), the transcriptional program is faithfully reactivated (Prasanth et al. 2003). This raises the important question of how the two daughter cells retain the memory of a defined gene expression program. Recent work has shown that a selective gene transcription program is maintained throughout mitosis (Palozola et al. 2017). However, how transcription is reactivated during mitotic exit and the underlying molecular mechanisms that control transcriptional memory remain poorly understood.

Several features of interphase chromatin landscapes remain associated with mitotic chromatin and have been proposed to function as “bookmarks” of transcriptional programs during mitosis. These “bookmarks” include retention of chromatin accessibility (Martínez-Balbás et al. 1995; Hsiung et al. 2015; Teves et al. 2016; Oomen et al. 2019), multiple general and lineage-specific TFs (Young et al. 2007; Kadauke et al. 2012; Caravaca et al. 2013; Deluz et al. 2016; Festuccia et al. 2016; Teves et al. 2016), and certain histone modifications (Kouskouti and Talianidis 2005; Valls et al. 2005; Muramoto et al. 2010; Zhao et al. 2011). Specific histone modification patterns can define distinct chromatin states of *cis*-regulatory elements (*cis*-REs), such as promoters and enhancers, and regulate gene expression via interaction with transcription factors. H3K4me3 and H3K27ac are highly enriched at active promoters near the transcription start site (TSS) and positively correlated with transcription. Enhancers play a crucial role in the activation and fine-tuning of their target promoters. Enhancer elements can exist in two major chromatin states, primed or active. Primed enhancers (PEs) are marked by H3K4me1/2 and their target genes are weakly or not expressed, whereas active enhancers (AEs) are additionally marked by the histone acetyltransferases CBP/p300-mediated H3K27ac and are associated with actively transcribed genes (Heintzman et al. 2007; Creyghton et al. 2010; Calo and Wysocka 2013). Recently, chromatin immunoprecipitation sequencing (ChIP-seq) studies revealed spatial genomic information of active histone marks, including H3K4me1/3, H3K9ac, or H3K27ac at prometaphase-arrested cells (Liang et al. 2015; Liu et al. 2017; Javasky et al. 2018). These studies allowed the comparison between mitotic and interphase histone marks and revealed specific modifications, dynamics of chromatin states at *cis*-REs, and estimation of TF binding that could serve as bookmarks. Although specific histone modifications remain associated with mitotic chromatin even at reduced levels, it remains unclear whether such association on mitotic chromatin has a regulatory role in transcriptional activation during or after mitosis. Furthermore, it remains to be determined whether histone modifications, retained either at promoters or enhancers, contribute as bookmarks of transcriptional program during mitosis.

Genome-wide chromosome conformation capture data, such as that generated by the Hi-C method, can reveal that the structural features of interphase chromosomes, such as A and B compartments, topologically associating domains (TADs), and DNA loops are lost during prometaphase and reestablished in G1 phase (Naumova et al. 2013; Abramo et al. 2019). TADs are thought of as important basic units of chromosome organization and are demarcated from each other by boundaries (Dixon et al. 2012; Nora et al. 2012). However, what mediates TAD formation after mitosis remains poorly understood. TAD boundaries in interphase have been shown to be enriched in active transcription, housekeeping genes, tRNA genes and short interspersed nuclear elements (SINEs), as well as binding sites for the architectural proteins CCCTC-binding factor (CTCF) and cohesin complex (Dixon et al. 2012). Furthermore, targeted degradation of CTCF, using the auxin-induced rapid degradation system, resulted in the almost complete elimination of TADs, suggesting CTCF's essential role for the establishment of TADs (Nora et al. 2017). It is currently unclear whether CTCF or other features enriched at the boundaries in interphase chromatin are also involved in the reestablishment of TADs after mitosis.

In this study, we combined histone modification ChIP-seq, EU-RNA-seq, and Hi-C from prometaphase to G1 phase to comprehensively examine on a genome-wide level the temporal relationship between histone modification binding, nascent transcription, and long-range chromatin interactions during the mitosis–G1 transition. We found that H3K4me3 remains associated with almost all promoters and that H3K4me1 is highly retained at enhancers of cell type-specific genes during prometaphase. In contrast, H3K27ac is largely reduced during prometaphase, although a subset of H3K27ac remains at enhancers of cell type-specific genes and promotes early expression of these cell type-specific genes. During the anaphase/telophase transition, most promoters gain H3K27ac, and this is essential for the accurate transmission of gene expression programs during cell division. Furthermore, TAD boundaries during prometaphase remain associated with H3K4me3 and H3K4me1 in the absence of CTCF, but CTCF is recruited and enriched at the boundaries during anaphase/telophase together with H3K27ac before the appearance of loops, TADs, and compartments. Altogether, the multilevel data of epigenetic landscape, nascent transcription, and TAD interaction shed light on not only the temporal order of genome organization after mitosis but also indicate a potential bookmarking role for histone modifications in accurate re-establishment of transcriptional activation pattern and long-range chromatin interactions.

## Results

### H3K27ac is lost in prometaphase and regained in anaphase/telophase

**Figure 1.**
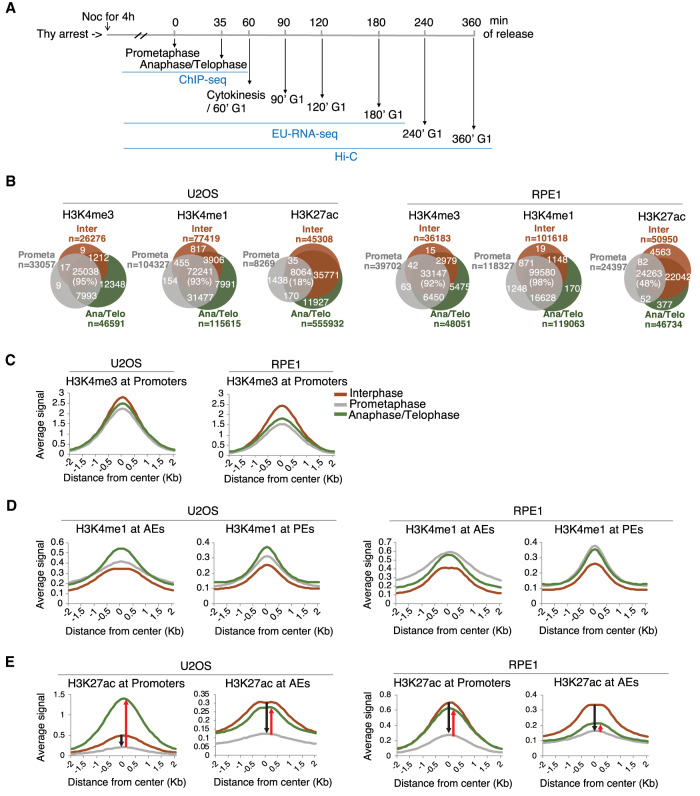
Cell cycle-dependent regulation of H3K27ac. (*A*) Schematic of the experimental strategy combining ChIP-seq, EU-RNA-seq, and Hi-C during prometaphase, anaphase/telophase, and various G1 phases. Mitotic cells were obtained by thymidine and nocodazole treatments. Anaphase/Telophase and G1 phase cells were obtained after release from mitotic arrest for indicated time. (*B*) Venn diagram showing the number of H3K4me3, H3K4me1, and H3K27ac binding sites that were found in interphase (Inter, orange), prometaphase (Prometa, gray), and anaphase/telophase (Ana/Telo, green) in U2OS (*left* panel) and RPE1 (*right* panel) cells. n represents the number of observed histone modification peaks in each cell cycle phase. Percentage of interphase peaks that were also detected in mitotic cells are shown. (*C*–*E*) Histogram showing ChIP-seq reads of H3K4me3 (*C*), H3K4me1 (*D*), and H3K27ac (*E*) ±2 kb surrounding the peak center of sites at promoters, AEs, or PEs during interphase, prometaphase, and anaphase/telophase in U2OS (*left* panel) and RPE1 (*right* panel). In *E*, black arrow indicates reduced ChIP-seq reads of H3K27ac from interphase to prometaphase at promoters and AEs. Red arrow indicates gained ChIP-seq reads of H3K27ac from prometaphase to anaphase/telophase at promoters and AEs.

Transcription is reactivated at the time of mitotic exit, as the NE starts reassembling and RNA Pol II globally rebinds to the chromatin (Prasanth et al. 2003). It is thus important to study this stage to fully understand genome organization during cell division. To determine the relationship between epigenetic dynamics, gene expression, and long-range chromatin interactions at the mitosis–G1 transition, we used three independent methods: (1) ChIP-seq analysis to identify genome localization mapping of key histone modifications, (2) EU-RNA-seq to capture temporal nascent transcription expression, and (3) Hi-C to determine the timing of long-range chromatin interactions from prometaphase to G1 phase (Fig. 1A). In order to obtain cells at different cell cycle stages including mitotic exit, we synchronized two human cell lines, osteosarcoma U2OS and retina pigment epithelia RPE1 cells using thymidine and nocodazole. The degree of synchrony at each cell cycle stage was monitored by fluorescence microscopy of Hoechst and α-Tubulin stained cells. Asynchronous populations (interphase) had <2% mitotic cells, nocodazole-arrested culture (prometaphase) contained >95% mitotic cells, and cells released from nocodazole-induced arrest for 35 min (anaphase/telophase) were ∼70% enriched for anaphase/telophase (Supplemental Fig. S1A). Thus, our synchronization approach enables us to collect enriched populations of cells at prometaphase, anaphase/telophase, and interphase, respectively.

To determine the mitotic retention of histone modifications in our system, we focused on three histone modifications, H3K4me3, H3K4me1, and H3K27ac, which are associated with the activation of transcription in promoters (H3K4me3, H3K27ac) or active enhancers (H3K4me1, H3K27ac). We performed immunofluorescence in asynchronous cells and Western blotting in histone extracts from interphase, prometaphase, and anaphase/telophase enriched cells. We observed that all three histone modifications (H3K4me3, H3K4me1, and H3K27ac) were detected in all phases of mitosis from both U2OS and RPE1 cells (Supplemental Fig. S1B,C). To comprehensively examine the extent and genomic localization of mitotic occupancy of histone modifications, we performed ChIP-seq for H3K4me3, H3K4me1, and H3K27ac in U2OS and RPE1 cell lines during interphase, prometaphase, and anaphase/telophase (Supplemental Table S1). When there is a global signal change under different experimental conditions (e.g., interphase vs. mitosis), typical normalization by the total number of mapped reads can be misleading since similar distributions of positions are sampled during sequencing. It is thus often challenging to quantitatively compare histone modification ChIP-seq data sets from different phases of the cell cycle. To overcome this, we used a spike-in based normalization strategy, whereby adding an equal amount of exogenous chromatin from a different species into each sample can be used as a more accurate normalization control (Orlando et al. 2014). We thus added spike-in chromatin from *Drosophila melanogaster* in each sample for global normalization and direct comparison of binding between interphase and mitotic sample (Egan et al. 2016). In accordance with previous observations (Liang et al. 2015; Javasky et al. 2018), our ChIP-seq analysis showed a significant overlap with binding sites of histone methylations between interphase and mitosis. We identified 26,276 H3K4me3 binding sites in interphase and 95% of those sites (25,038) were retained on chromatin during mitosis in U2OS and 92% of sites in RPE1. Similarly, 93% of interphase H3K4me1 binding sites in U2OS and 98% of H3K4me1 sites in RPE1 were detected in mitosis. In contrast, consistent with previous observations (Zhiteneva et al. 2017; Ginno et al. 2018; Javasky et al. 2018), H3K27ac showed a reduction in mitosis in both U2OS and RPE1. Only 18% of H3K27ac interphase binding sites in U2OS and 48% in RPE1 remained in mitosis (Fig. 1B). Therefore, our spike-in normalized ChIP-seq enables us to quantitatively compare the genomic localization of histone modifications during mitosis–G1.

We next asked whether the genomic distribution of histone modifications between interphase and mitosis appears to be uniform or different at active regulatory elements. We first classified promoter (H3K4me3^+^, proximity to TSS), PE (H3K4me1^+^, distal to TSS), and AE (H3K27ac^+^/H3K4me1^+^, distal to TSS) elements based on our ChIP-seq data (Supplemental Fig. S1D; Creyghton et al. 2010; Calo and Wysocka 2013). We then compared histone modification levels across the cell cycle at these elements. As has been shown before, interphase H3K4me3 peaks were preferentially bound at TSS (Supplemental Fig. S1E, left panel) and H3K4me1 peaks were depleted at TSS (Supplemental Fig. S1E, right panel; Heintzman et al. 2009; Creyghton et al. 2010). During mitosis, H3K4me3 binding was significantly enriched, comparable with interphase at promoters (Fig. 1C). Mitotic H3K4me1 was also detected at a similar level in interphase, but with slightly higher levels at AEs and PEs (Fig. 1D). In contrast, we observed signal reduction in H3K27ac levels in the prometaphase cells at both promoters and AEs and its signals recovered in anaphase/telophase. The reduction of H3K27ac binding was observed in both promoters and AEs with similar levels, however the recovery of binding in anaphase/telophase was more pronounced at promoters (Fig. 1E), suggestive of the promoter-specific function of H3K27ac during anaphase/telophase. Altogether, these results demonstrate that, based on the number of binding sites and binding distribution at *cis-*REs of histone modifications, H3K4me3 and H3K4me1 are globally retained during mitosis, while H3K27ac is lost in prometaphase and regained as cells exit mitosis.

### Promoters maintain H3K4me3 and enhancers maintain H3K4me1 in the absence of H3K27ac during prometaphase

**Figure 2.**
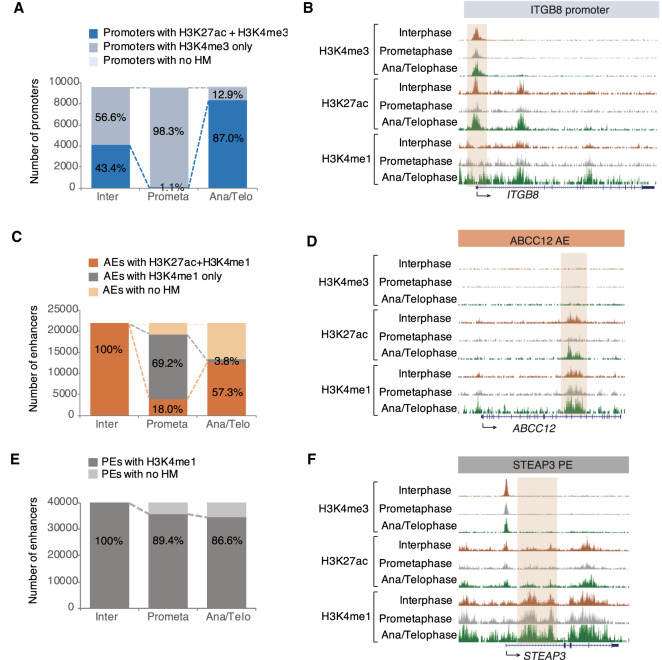
Histone methylations bookmark promoters and enhancers in the absence of H3K27ac during prometaphase. (*A*) Quantification of interphase promoters that are also detected in prometaphase or in anaphase/telophase. Promoters harboring both H3K27ac and H3K4me3 are shown in blue and H3K4me3 only in light blue. Promoters not containing any histone modification are shown in the lightest blue color. (*B*) ChIP-seq tracks at the promoter of the *ITGB8* gene for H3K4me3, H3K27ac, and H3K4me1 during interphase, prometaphase, and anaphase/telophase. (*C*) Quantification of interphase AEs that are also detected in prometaphase or in anaphase/telophase. AEs harboring both H3K27ac and H3K4me1 are shown in orange and H3K4me1 only in gray. AEs not containing any histone modification are shown in light orange. (*D*) ChIP-seq tracks at AE of *ABCC12* gene for H3K4me3, H3K27ac, and H3K4me1 during interphase, prometaphase, and anaphase/telophase. (*E*) Quantification of interphase PEs that are also detected in prometaphase or in anaphase/telophase. PEs harboring H3K4me1 are shown in gray. PEs not containing any histone modification are shown in light gray. (*F*) ChIP-seq tracks at PE of *STEAP3* gene for H3K4me3, H3K27ac, and H3K4me1 during interphase, prometaphase, and anaphase/telophase. Peaks are highlighted by brown boxes. (Inter) Interphase; (Prometa) prometaphase; (Ana/telo anaphase/telophase); (HM) histone modification.

We next wanted to determine how gene regulatory elements “remember” their histone modification dynamics across mitosis. To answer this question, we selected all promoters and enhancers from interphase and compared the dynamics of each histone modification from interphase to prometaphase or to anaphase/telophase cells. Promoters can be marked by either H3K4me3 only or by both H3K27ac and H3K4me3. We found that the majority of H3K4me3+ promoters remained in prometaphase (98.3%). In contrast, only 1.1% of promoters remained associated with H3K27ac in prometaphase and 87% of H3K4me3+ promoters were able to regain H3K27ac in anaphase/telophase (Fig. 2A,B; Supplemental Fig. S2A,B). Only a small fraction of interphase AEs retained H3K27ac in prometaphase (18.0%). Of AEs, 69.2% lost H3K27ac, but retained H3K4me1 in prometaphase cells (Fig. 2C,D; Supplemental Fig. S2C,D). We found that the majority of PEs remained stable in prometaphase and anaphase/telophase (89.4% and 86.6%, respectively) similar to H3K4me3^+^ promoters (Fig. 2E,F; Supplemental Fig. S2E,F). These data suggest that promoters and enhancers continue to retain H3K4me3 and H3K4me1 respectively, as potential bookmarks, although they lose H3K27ac during prometaphase.

### H3K4me1 remains at enhancers of cell type-specific genes during prometaphase

**Figure 3.**
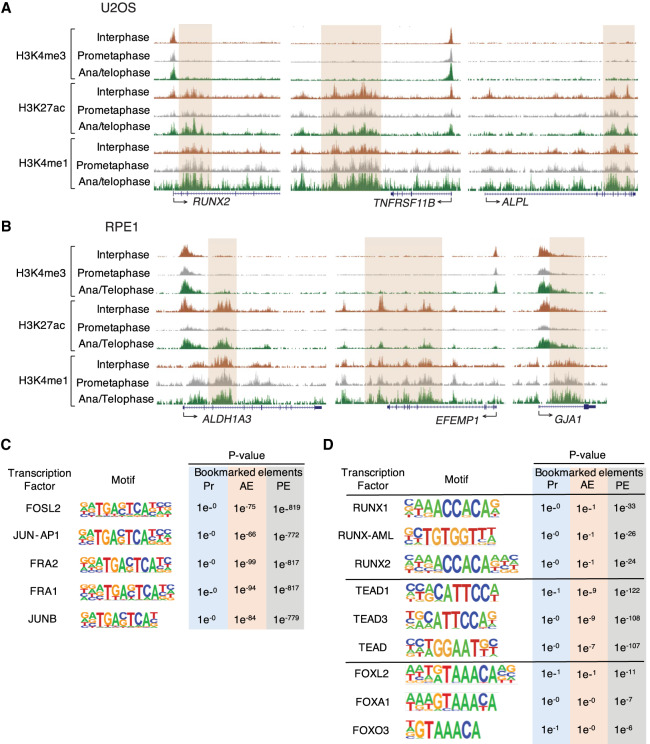
H3K4me1 remains at enhancers of cell type-specific genes during prometaphase. (*A*) Representative genome browser tracks showing cobinding of osteoblast-specific genes (*RUNX2*, *TNFRSF11B*, and *ALPL*) with mitotic H3K4me1 at enhancers. (*B*) Representative genome browser tracks showing cobinding of RPE-specific genes (*ALDH1A3*, *EFEMP1*, and *GJA1*) with mitotic H3K4me1 at enhancers. Peaks are highlighted by brown boxes. (*C*) TF motif enrichments for bookmarked promoter (Pr, blue), AE (orange), and PE (gray) during mitosis. Sequence logos and *P* values are shown for the most highly enriched sequence motifs among the top 10. (*D*) Motif analysis of TFs-regulating osteoblast differentiation (RUNXs, TEADs, and FOXOs) for bookmarked promoter (Pr, blue), AE (orange), and PE (gray).

Next, we asked whether continuous retention of H3K4me1 during prometaphase associates with a bookmark of cell type-specific genes. For this, we used U2OS cells, which are derived from cells of mesenchymal origin that differentiate to osteoblasts. There are several genes identified as essential for bone formation and differentiation to osteoblasts. These so-called osteoblast-specific genes include *RUNX2*, *TNFRSF11B*, *DKK1*, *SP7*, *ALPL*, *MSX2*, *COL1A2*, and *SOST* (Kirkham and Cartmell 2007; Chapurlat and Confavreux 2016; Rutkovskiy et al. 2016). Strikingly, all of these genes were associated with mitotic H3K4me1 at enhancers (*RUNX2*, *TNFRSF11B*, and *ALPL* shown in Fig. 3A). Consistent with U2OS cells, RPE1 cells also showed that H3K4me1 during prometaphase was associated with RPE-specific genes (Zhang et al. 2012) such as *ALDH1A3*, *EFEMP1*, *GJA1*, *TIMP3*, *AHR*, *TYRP1*, *LAMP2*, *SLC16A4*, *BMP4*, *VEGFA*, and *PRNP* (*ALDH1A3*, *EFEMP1*, and *GJA1* shown in Fig. 3B). Next, we wanted to investigate whether mitotic histone modification binding sites are linked to genes with distinct biological functions. To do so, we performed Genomic Regions Enrichment of Annotations Tool (GREAT) (McLean et al. 2010) analysis on the promoters and enhancers in U2OS and RPE1 cells (Supplemental Fig. S3; Supplemental Table S2). Notably, promoters retaining H3K4me3 during prometaphase, were strongly enriched for genes involved in fundamental cellular processes, such as RNA and protein metabolism, whereas AEs either retaining or losing H3K27ac during prometaphase were predominantly linked to differentiation or development-related genes in both cell lines. Specifically, we observed that AEs losing H3K27ac, but retaining H3K4me1 were most strongly associated with genes involved in bone mineralization in U2OS (Supplemental Fig. S3A, bottom panel). Together, these results indicate that enhancers use H3K4me1 to maintain their cellular identity upon loss of H3K27ac throughout mitosis.

Previous studies demonstrated that although most TFs and cofactors are removed from mitotic chromatin, several tissue-specific or pathway-specific TFs such as RUNX2, GATA1, or FOXA1 remain bound on mitotic chromosomes to a subset of their interphase sites (Young et al. 2007; Kadauke et al. 2012; Caravaca et al. 2013). To determine the factors that associate with mitotic histone modifications at *cis*-REs, we performed de novo motif analysis of the bookmarked elements in U2OS cells. Our analysis showed that both AEs and PEs were preferentially enriched with motifs of the activator protein-1 (AP-1) family of TFs including FOSL2, JUN-AP1, FRA2, FRA1, and JUNB (Fig. 3C), which has an essential role in osteoblast differentiation and collagen production by regulating osteoblast-lineage gene expression in bone cells (Jochum et al. 2000; Sabatakos et al. 2000; Kveiborg et al. 2004; Bozec et al. 2010). In addition to AP-1 TFs, we next sought to determine whether genomic regions harboring mitotic histone marks were enriched for TFs binding that has been known to regulate osteoblast differentiation such as RUNXs (Komori 2005), TEADs (Kegelman et al. 2018), and FOXOs (Rached et al. 2010; Teixeira et al. 2010). Our analysis revealed that these TFs regulating osteoblast-specific gene expression had preferential binding at PEs compared with AEs and promoters (Fig. 3D). A prior study shows that RUNX2, an essential TF for osteogenic cell fates, remains associated with mitotic chromosomes in multiple cell lines, including Saos-2 osteosarcomas and HeLa cells (Young et al. 2007). Therefore, combined with previous observations, we conclude that retention of mitotic H3K4me1 peaks at enhancer regions provides a platform for interaction with a subset of cell type-specific TFs during and after mitosis.

### Regained H3K27ac in anaphase/telophase is positively correlated with gene reactivation

**Figure 4.**
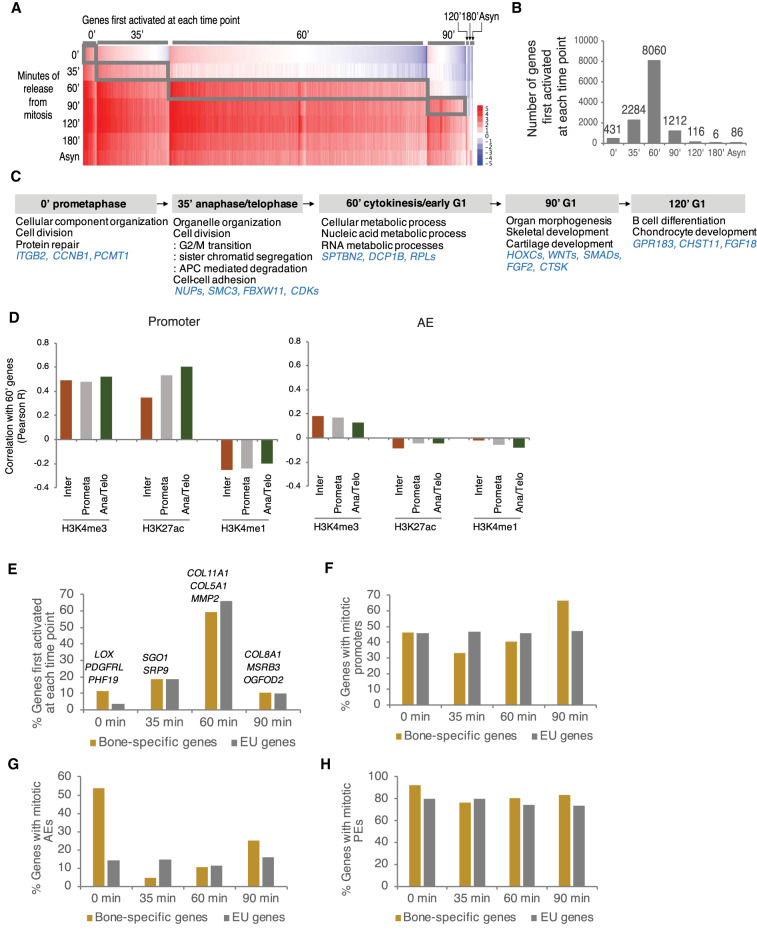
Activation of transcriptional programs during mitosis–G1 is associated with histone modification binding. (*A*) EU-RNA-seq data heat map illustrating the relative expression of genes first activated at each time point during mitosis–G1. (*B*) Number of EU labeled genes first activated at each time point. (*C*) Representative GO terms at each time point. (*D*) Pearson correlation coefficient (R) was determined between 60-min transcriptional level and the ChIP-seq reads of H3K4me3, H3K27ac, and H3K4me1 in interphase (Inter), prometaphase (Prometa), and anaphase/telophase (Ana/Telo) at promoter and AE, defined in Supplemental Figure S1D. (*E*–*H*) Bar plot showing the percentage of genes first activated at each time point. (*E*) Representative bone-specific genes are indicated at each time point. Percentage of genes containing mitotic promoters (*F*), mitotic AEs (*G*), and mitotic PEs (*H*) at each time point. Bone-specific genes are shown in yellow and EU genes are in gray.

Histone modifications are found on mitotic chromatin with either a stable (H3K4me1/3) or reduced (H3K27ac) abundance when compared with interphase chromatin (Fig. 1). Together with an association with mitotic chromatin, it has been suggested that one feature of mitotic bookmarking is to enable activation of a defined transcriptional program after mitosis (Kouskouti and Talianidis 2005; Valls et al. 2005; Muramoto et al. 2010; Zhao et al. 2011). To determine whether occupancy of mitotic histone modifications associates with postmitotic transcriptional activation, we first examined temporal dynamics of gene expression during the mitosis–G1 transition. To do that, we labeled nascent transcripts with uridine analog, 5′-ethynyl uridine (EU) for 35 min, prior to isolation of RNA, during prometaphase, anaphase/telophase, cytokinesis, and various G1 phases (Supplemental Fig. S4A). Then, we captured nascent RNA by adding biotin-azide and generated cDNA libraries for sequencing. As there was a large difference in the amount of nascent RNA between interphase and mitotic cells, we used external controls that appropriately normalize the RNA-seq data (Supplemental Fig. S4B; Supplemental Table S1; Palozola et al. 2017). Based on normalized FPKM, genes were divided into seven classes, first activated at 0 min, 35 min, 60 min, 90 min, 120 min, 180 min, and asynchronous (Supplemental Table S3). Consistent with the previous study, we found a hierarchy of gene reactivation at the mitosis to G1 transition (Fig. 4A; Palozola et al. 2017). We also found a subset of genes expressed in mitosis (431 genes at 0 min and 2284 genes at 35 min) and the largest number of genes first activated at 60 min when most of the cells are at cytokinesis (Fig. 4B). Gene Ontology (GO) enrichment analysis showed that genes first activated at 60 min are involved in basic cellular functions such as cellular organization, cell cycle progression, and RNA and protein metabolism. Conversely, genes expressed at later time points are linked to bone development (Fig. 4C). However, these cell type-specific genes may be expressed at all times, but just overrepresented at the later time points, given that genes related to basic functions are first activated at the earlier time point by 60 min, when the majority of genes (88%) are expressed.

Next, we asked whether the presence of investigated histone modifications correlates with transcriptional activation during and after mitosis. To do that, we tested the Pearson correlation coefficient between transcription level of genes first activated at each time point and the read counts for histone modifications, at promoters versus AEs, and in each cell cycle phase. We found that at promoters, gene expression at 60 min was positively correlated with H3K4me3 or H3K27ac levels throughout all cell cycle phases. Notably, among the positive correlations, we found that genes whose promoters were associated with anaphase/telophase H3K27ac were most strongly correlated (*R* = 0.60) (Fig. 4D). Furthermore, we observed that all genes first activated at each time point showed the most positive correlation with anaphase/telophase H3K27ac at promoters (Supplemental Fig. 4C). AEs are associated with active transcription by bringing DNA loops physically close to gene promoters. However, for AEs, either H3K27ac or H3K4me1 level in mitosis was not positively correlated with gene expression (Fig. 4D). Altogether, these results indicate that recovery of H3K27ac at promoter regions in anaphase/telophase may be important for the reactivation of transcriptional programs in the daughter cells.

We observed that H3K4me1 at enhancers remains associated with cell type-specific genes during prometaphase (Fig. 3C; Supplemental Fig. S3). We thus asked whether H3K4me1 occupancy throughout mitosis associates with rapid transcriptional activation of cell type-specific genes. We obtained the list of 113 genes specifically expressed in bone or bone marrow, which are also expressed in U2OS, using the Tissue-specific Gene Expression and Regulation (TiGER) database (Liu et al. 2008). This number was similar to the 149 liver-specific genes in HUH7 human hepatoma cells seen in a prior study (Palozola et al. 2017). We then analyzed the time when the 113 bone-specific genes are first activated across mitosis and found that bone-specific genes follow general gene expression pattern: Most are reactivated at 60 min. Interestingly, however, there was a higher percentage of bone-specific genes first activated at 0 min, compared with EU-labeled total genes (Fig. 4E). To investigate whether bone-specific genes expressed at 0 min tend to contain mitotic histone marks at enhancers, the number of bone genes was counted at each time point based on its occupancy with either promoters retaining H3K4me3 or enhancers retaining H3K27ac/H3K4me1 during prometaphase. Strikingly, mitotic AEs and PEs showed higher enrichment for 0 min expressing bone-specific genes than EU-labeled total genes (Fig. 4G,H), while mitotic promoters did not show any particular enrichment for 0 min expressing bone-specific genes (Fig. 4F). This suggests that activation of early expressing bone-specific genes may involve association with mitotic H3K4me1 together with H3K27ac at enhancers.

### H3K27ac functions as a transcriptional bookmark

**Figure 5.**
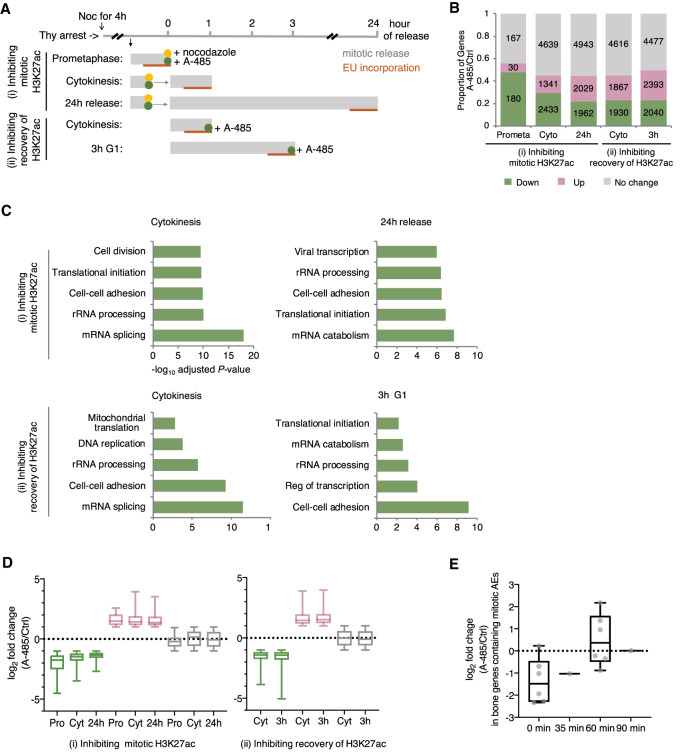
H3K27ac serves as a mitotic bookmark. (*A*) Schematic of EU-RNA-seq strategy illustrating strategies to test bookmarking function. A-485 was used to inhibit H3K27ac binding during and after mitosis. (*B*) Proportion of genes down-regulated or up-regulated, or with no change due to A-485 treatment in each cell cycle phase. The number of each group of genes is indicated. Down-regulated genes (green, log_2_ fold change < −1); Up-regulated genes (red, log_2_ fold change > 1). (*C*) Top five GO enriched for down-regulated genes in A-485 treated cells. Bar length represents the −log_10_ adjusted *P-*value. (*D*) Box and whisker plots of the log_2_ fold change in each cell cycle phase on treatment with A-485 across down-regulated, up-regulated, and no change genes. Midline equals the median, top of box equals 75th percentile, and bottom of box equals 25th percentile. Upper and lower whiskers equal the maximum and the minimum, respectively. (*E*) Box plot displaying the log_2_ fold change in expression of bone genes containing mitotic AEs at each time point upon A-485 treatment. Each gene is indicated with a dot (gray).

Mitotic occupancy of histone modifications is associated with postmitotic gene activation suggestive of mitotic bookmarking. However, a recent study showed that inhibition of mitotic BRD4 binding did not affect transcriptional activation, suggesting that mitotic occupancy can be passively bound to mitotic chromatin and dispensable for transcription (Behera et al. 2019). To test whether H3K27ac has a functional bookmarking role in postmitotic activation of transcription, we examined how inhibition of H3K27ac affects gene expression. We used the p300/CBP catalytic inhibitor A-485 (Lasko et al. 2017) to inhibit (1) mitotic H3K27ac occupancy or (2) recovery of H3K27ac from anaphase/telophase. First, we observed by Western blot that 1-h treatment of A-485 effectively reduced H3K27ac binding from nocodazole-arrested prometaphase cells (Supplemental Fig. S5A, left panel). We then washed out both nocodazole and A-485 and released cells for 1 h or 24 h. Reduced H3K27ac binding from prometaphase chromatin was able to regain as cells exit mitosis to enter 1-h cytokinesis or 24-h next cell cycle (Supplemental Fig. S5A, middle panel). Second, to inhibit H3K27ac regaining from anaphase/telophase, we released cells from mitotic arrest while treating with A-485 for 1 or 3 h. The H3K27ac levels successfully decreased in both 1-h and 3-h treatment with A-485 (Supplemental Fig. S5A, right panel). Next, nascent transcripts were pulse-labeled with the EU followed by sequencing and normalized by spike-in controls (Fig. 5A; Supplemental Table S1). We confirmed by EU-RNA-seq analysis that the biological replicates for each treatment were highly correlated (Supplemental Fig. S5B) and that A-485 treatment resulted in extensive transcriptional changes for all treatments (Supplemental Fig. S5C).

We first explored whether A-485 treatment alters postmitotic transcriptional activation. Genes were clustered into three groups based on log_2_ fold change (down-regulated, up-regulated, and no change) upon A-485 treatment. When inhibiting mitotic H3K27ac, 56% (48% down and 8% up) of the genes in prometaphase and 45% (29% down and 16% up) of the genes in cytokinesis were dysregulated. Since the length of the cell cycle is ∼24 h for dividing mammalian cells, we let cells enter the next cell cycle upon mitotic inhibition of H3K27ac. Surprisingly, 45% (22% down and 23% up) of the genes in 24-h release cells were dysregulated, suggesting that mitotic H3K27ac is required for maintaining transcriptional memory. H3K27ac begins to get regained from anaphase/telophase (Fig. 1) and is the most predictive histone mark for gene reactivation (Fig. 4D), suggestive of a transcriptional bookmark. We thus investigated the functional role of H3K27ac recovery from anaphase/telophase in mitotic bookmarking. When inhibiting recovery of H3K27ac, 45% (23% down and 22% up) of the genes in cytokinesis were dysregulated and 50% (23% down and 27% up) of the genes in 3 h G1 were also dysregulated (Fig. 5B). This suggests that postmitotic transcriptional activation requires the recovery of H3K27ac. GO enrichment analysis showed that genes down-regulated in all A-485-treated cells after cytokinesis were significantly enriched in gene sets involved in mRNA/protein metabolism and cell–cell adhesion (Fig. 5C), supporting the idea that faithful transmission of transcriptional programs requires both mitotic H3K27ac occupancy and H3K27ac recovery from anaphase/telophase.

Since transcription rate gradually increases as cells exit mitosis to enter the G1 phase (Fig. 4A), we hypothesized that perturbation of transcriptional activation by A-485 treatment would get recovered as cells get into late G1 (3-h G1) from early G1 (cytokinesis). However, we noticed that the extent of down-regulated and up-regulated genes was consistent between cells in cytokinesis and 3-h G1 upon A-485 treatment (Fig. 5D, right panel). The extent of down-regulated or up-regulated genes was also consistent between cells in cytokinesis and 24-h release upon inhibition of mitotic H3K27ac (Fig. 5D, left panel). Therefore, the dysregulated genes by both inhibiting mitotic H3K27ac and H3K27ac recovery were not able to recover their own transcriptional activity in the daughter cells. These data suggest that H3K27ac during prometaphase and anaphase/telophase is essential for the activation of postmitotic transcription, rather than regulates transcriptional rate.

Given that bone-specific genes expressed early tend to contain mitotic H3K27ac (Fig.4G), we hypothesized that removal of H3K27ac from mitotic chromatin would affect the early activation of bone-specific genes. Indeed, A-485 treatment during mitosis altered expression in bone-specific genes. Bone-specific genes at the 0 min timepoint were most noticeably down-regulated upon mitotic inhibition of H3K27ac (Fig. 5E). This indicates that mitotic H3K27ac is required for rapid transcriptional activation of cell type-specific genes.

### Long-range chromatin interactions are established between 90 and 120 min after release from mitotic arrest

**Figure 6.**
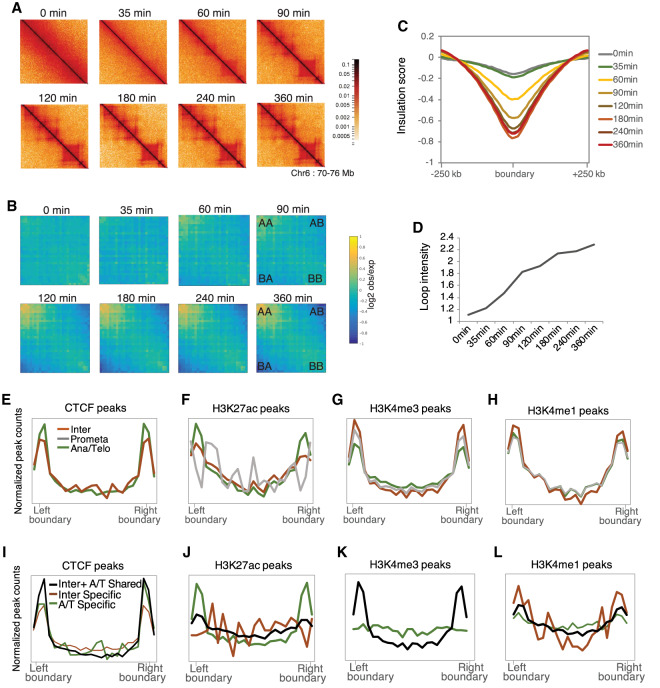
Long-range chromatin interactions are reformed between 90 and 120 min after release from mitotic arrest. (*A*) Hi-C contact maps of chromosome 6: 70,000,000–76,000,000 showing TADs for each time point. (*B*) Saddle plots showing global A-A, B-B, and A-B compartment signals for each time point. A/B compartment score derived from first principle components were sorted and divided into 30 quantiles. Saddle plots show the average observed/expected interaction frequency between regions according to the eigen vector values of both ends. (*C*) Average insulation score near TAD boundaries for each time point. (*D*) Average DNA loop intensity for each time point. Loop annotations were firstly identified for the 360-min time point using hiccups. The loop intensity is the average observed/expected interaction frequency. (*E*–*H*) ChIP-seq signals of CTCF (*E*), H3K27ac (*F*), H3K4me3 (*G*), and H3K4me1 (*H*) in interphase (orange), prometaphase (gray), and anaphase/telophase (green) at TAD boundary regions. (*I*–*L*) Interphase and anaphase/telophase peaks were categorized into either interphase-specific (Inter Specific, orange), anaphase/telophase-specific (A/T Specific, green), or shared between interphase and anaphase/telophase (Inter + A/T Shared, black). CTCF (*I*), H3K27ac (*J*), H3K4me3 (*K*), and H3K4me1 (*L*) peaks in each category were aligned at TAD boundary regions. In *K*, there were no H3K4me3 interphase-specific peaks that were observed at TAD boundary regions.

Long-range chromatin interactions are lost in prometaphase and reformed in early G1 (Naumova et al. 2013; Abramo et al. 2019). However, the role of TADs interactions during anaphase/telophase and whether they are already reestablished during anaphase/telophase to enable gene reactivation at cytokinesis remain unclear. To investigate when long-range interactions are reformed, we performed Hi-C to detect physical interactions between distant genomic elements, from prometaphase to 360-min G1 (Supplemental Table S1). We assessed the reproducibility of Hi-C data between two biological replicates and observed a strong Pearson correlation coefficient at all time points (Supplemental Fig. S6). In agreement with previous observations, Hi-C contact maps showed that chromosomal interactions were no longer observed in mitotic cells (Naumova et al. 2013; Nagano et al. 2017; Gibcus et al. 2018; Oomen et al. 2019) including 0-min (prometaphase) and 35-min (anaphase/telophase) chromatin and appeared after 60 min (cytokinesis) (Fig. 6A; Abramo et al. 2019). We observed that A-A and B-B compartments, TADs, and DNA loops between promoters and enhancers were no longer detected in prometaphase and started to reveal interactions from 60 min and completed between 90 and 120 min (Fig. 6B–D). Because long-range interactions are reformed after 60 min when the transcriptional program is reestablished, we reasoned that the formation of long-range interactions does not appear to be necessary for the majority of gene reactivation.

### CTCF and active histone modifications bookmark the TAD boundaries across mitosis

It has been shown previously that the TAD boundaries are enriched in CTCF binding (Dixon et al. 2012; Rao et al. 2014; Vietri Rudan et al. 2015) and imaging analysis detects CTCF on mitotic chromatin, yet with reduced levels (Burke et al. 2005). To investigate how properly TADs are formed after mitosis, we asked whether a subset of CTCF is retained at the TAD boundaries across mitosis and acts as a bookmark for TAD formation. To do that, we first performed ChIP-seq for CTCF in prometaphase, anaphase/telophase, and interphase and analyzed retention of CTCF binding and its genome-wide distribution during mitosis. In accordance with a recent study, CTCF is largely depleted from mitotic chromatin (Oomen et al. 2019). We identified <3% of the total binding peaks in prometaphase (Supplemental Fig. S7C). In concordance with our ChIP-seq analysis, immunofluorescence and Western blot analyses showed that CTCF is largely dispersed to the cytoplasm in prometaphase, then recruited on chromatin in anaphase/telophase (Supplemental Fig. S7A,B). We then analyzed the distribution of CTCF binding peaks at the TAD boundaries in each cell cycle phase. As previously suggested, interphase CTCF binding was enriched at the TAD boundaries. We did not observe any enrichment at the boundaries in prometaphase, possibly due to the limited numbers of binding sites during this stage. Strikingly, anaphase/telophase CTCF binding was strongly enriched at the boundaries even higher than that seen in interphase (Fig. 6E). These data indicate that although CTCF is not responsible for “memory” of insulator elements during prometaphase, it may play a role in TAD reformation during anaphase/telophase, particularly by relocalizing at the boundaries.

Given that active histone marks are enriched at the TAD boundaries in the interphase chromatin (Dixon et al. 2012) and H3K4me3 and H3K4me1 are well retained throughout mitosis at *cis*-REs (Fig. 2), we hypothesized that H3K4me3 and H3K4me1 may bookmark the TAD boundaries in prometaphase when CTCF is absent. We examined the distribution of histone modification binding sites at the TAD boundaries in interphase, prometaphase, and anaphase/telophase. As expected, mitotic H3K27ac was depleted at the boundaries as reduced in prometaphase (Fig. 6F). Strikingly, however, H3K4me3 and H3K4me1 were enriched at the boundaries in all cell cycle phases including prometaphase and anaphase/telophase (Fig. 6G,H), suggesting that H3K4me3 and H3K4me1 act as a bookmark of insulator elements to allow cells to remember where TADs need to be formed after mitosis.

Although CTCF and H3K27ac binding were significantly decreased in prometaphase, 56% of CTCF (17,004 peaks) (Supplemental Fig. S7C) and 79% of H3K27ac (35,771 peaks) (Fig. 1B) interphase binding sites were recovered in anaphase/telophase. Interestingly, we found new binding sites on anaphase/telophase chromatin for CTCF (1728 peaks) and H3K27ac (11,927 peaks). In addition, both CTCF and H3K27ac were strongly enriched at the TAD boundaries, especially in anaphase/telophase (Fig. 6E,F). Thus, we investigated whether CTCF or H3K27ac may have anaphase/telophase-specific function on insulator elements. To this end, peaks were divided into three categories: (1) “shared,” observed in both interphase and anaphase/telophase; (2) “interphase-specific,” observed only in interphase; and (3) “anaphase/telophase-specific,” observed only in anaphase/telophase. When characterizing the distribution of binding peaks in each category at the boundaries, we observed that CTCF “shared” peaks were highest at the boundaries (Fig. 6I), indicating that CTCF is recruited at the boundaries in anaphase/telophase and remains there in the next G1 phase. Conversely, H3K27ac “anaphase/telophase-specific” peaks were greatly enriched at the boundaries, stronger than “shared” peaks, while “interphase-specific” peaks were depleted (Fig. 6J), indicating that enrichment of H3K27ac binding at insulators is mostly either newly bound to anaphase/telophase or recovery of interphase binding after prometaphase. We did not observe noticeable enrichment in either H3K4me3 or H3K4me1 “anaphase/telophase-specific” binding (Fig. 6K,L). Together, these results suggest that the increase in H3K27ac binding during anaphase/telophase may contribute to TAD reformation.

## Discussion

**Figure 7.**
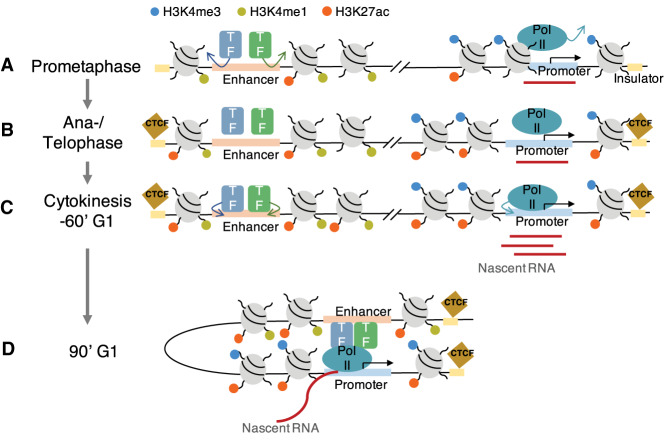
Temporal regulation of genome reorganization by histone modifications, nascent transcription, and long-range chromatin interactions during mitosis–G1. (*A*) In prometaphase, Pol II and TFs, as well as CTCF, dissociate, although some genes are actively transcribed. H3K27ac is globally lost except for a subset of enhancers of cell type-specific genes and limited promoters of housekeeping genes, while H3K4me3 and H3K4me1 are stably bound at promoter and enhancer respectively, and both at insulator. (*B*) In anaphase/telophase, H3K27ac is recruited back at promoter, enhancer, and insulator. CTCF is also recruited at insulator. (*C*) In cytokinesis/60-min G1, Pol II and TFs reassociate, and most genes are reactivated. (*D*) In 90-min G1, long-range chromosomal interactions appear to be reestablished.

Here, we provide a temporal order of genome organization during and after mitosis. During prometaphase, histone methylation binding is largely retained, however only low levels of transcription occur and no long-range chromatin interactions are detected. Because histone modifications are established first, we investigated whether mitotic histone modification binding contributes to gene reactivation or to the formation of long-range chromatin interactions. We observed that levels of H3K4me3 and H3K4me1 are stable throughout mitosis at promoters and enhancers, respectively, and both at insulators. H3K4me1 remains at enhancers of cell type-specific genes and TFs binding motifs, which regulate cell type-specific gene expression during prometaphase. This indicates that histone methylations act as a widespread epigenetic memory of regulatory information previously active in the mother cells. Limited H3K27ac remains associated with mitotic chromatin for the accurate transmission of transcription programs and early expression of cell type-specific genes. Loss of H3K27ac in prometaphase is recovered in anaphase/telophase preferentially at promoters and insulators. This recovery of H3K27ac by anaphase/telophase is required for gene reactivation and is particularly relocalized at the TAD boundaries. When cells enter cytokinesis, most of the genes are transcribed. Long-range chromatin interactions gradually increase from cytokinesis, and are complete between 90 and 120 min G1. In addition to H3K27ac, CTCF lost in prometaphase is recruited in anaphase/telophase at the TAD boundaries, which may be involved in the reformation of TADs in the daughter cells. Overall, the genome is orderly reformed in which histone methylations are retained throughout mitosis, followed by histone acetylation and CTCF in anaphase/telophase, transcription in cytokinesis, then long-range interactions in 90 min G1 (Fig. 7).

Consistent with previous reports using different approaches such as quantitative mass spectrometry (Zhiteneva et al. 2017; Ginno et al. 2018; Javasky et al. 2018), our ChIP-seq analysis reveals the global retention of H3K4me3 and H3K4me1, but reduction in H3K27ac during prometaphase. Our spike-in normalized ChIP-seq allows us to study quantitative and spatial changes in histone modifications during the mitosis–G1 transition. This retention of histone modifications has been suggested to function as a bookmark of the transcriptional program during mitosis. Notably, we observed that widespread histone methylations on mitotic chromatin bookmark *cis*-REs. First, H3K4me3 bookmarks promoters of genes during prometaphase. Ninety-eight percent of promoters retain H3K4me3 during prometaphase (Fig. 2A) and mitotic H3K4me3 binding has positive correlations with gene expression (Fig. 4D). However, harboring H3K4me3 does not seem to facilitate rapid transcriptional reactivation because the Pearson correlation between H3K4me3 signals and gene expression is not particularly high in early expressing genes such as 0-min or 35-min genes, compared with late expressing genes such as 90-min, 120-min, or 180-min genes (Supplemental Fig. S4C). Second, H3K4me1 bookmarks enhancers of cell type-specific genes during prometaphase. Cell type-specific genes tend to contain H3K4me1 during prometaphase and to be expressed early in the mitosis–G1 transition (Figs. 3A,B, 4H). This may associate with TFs binding such as AP1 family TFs (Long 2012), RUNX2 (Ducy et al. 1997; Komori et al. 1997; Otto et al. 1997), TEADs (Kegelman et al. 2018), or FOXO1 (Rached et al. 2010; Teixeira et al. 2010), which are known to have a crucial role in bone formation or homeostasis. Genome-wide approach remains to be examined to reveal whether these TFs occupy with H3K4me1 during mitosis at cell type-specific enhancers. Last, H3K4me3 and H3K4me1 bookmark insulators during prometaphase (Fig. 6G,H). Although active histone marks have been well investigated to be enriched at TAD boundaries, the distribution of mitotic histone marks at TADs boundaries has not. Altogether, these data indicate that mitotic H3K4me3 and H3K4me1 contribute to the faithful establishment of the transcriptional program, epigenetic landscapes, and chromatin architecture across mitosis. Similar to these active histone methylation marks, repressive histone methylation marks, including H3K9me2, H3K9me3, and H3K27me3, are globally maintained during mitosis (Wang and Higgins 2013). A recent study shows that H3K9me2 ensures the inheritance of spatial position at the nuclear periphery throughout mitosis (Poleshko et al. 2019). Thus, these suggest that repressive histone methylation marks likewise would have a role in epigenetic memory of transcriptionally silent genes as well as spatial organization of genome throughout mitosis.

In our ChIP-seq analysis, H3K27ac substantially increases in the number of binding sites and binding signal on anaphase/telophase chromatin (Fig.1B,E). First, this increase may have a role in transcriptional reactivation. A recent study shows that H3K27ac signals during mitotic exit correlate with increased transcriptional activity in early G1 at both promoters and enhancers by measuring Pol II binding in erythroblasts (Hsiung et al. 2016). Our analysis combined with EU-RNA-seq, and with the use of spike-in normalization, reveals that H3K27ac in anaphase/telophase, deposited only at promoters but not at enhancers, is also the most predictive histone mark for transcriptional reactivation among the three active histone marks tested in this study (Fig. 4D). Enhancer-promoter loops were lost in mitosis and not formed until cytokinesis at 60 min (Fig. 6D), which may explain why we did not observe any positive correlations with transcriptional activation at AE regions. Furthermore, A-485-treated H3K27ac inhibition confirmed that H3K27ac recovery has a mitotic bookmarking function. Surprisingly, transcriptional activation was perturbed for ∼50% of dysregulated genes by inhibiting H3K27ac recovery (Fig. 5B), indicating that H3K27ac is required for posttranscriptional activation. In addition to transcription, as H3K27ac is enriched at the TAD boundaries in interphase chromatin (Dixon et al. 2012), we tested whether the increase of H3K27ac in anaphase/telophase has a role in the formation of TADs. We found a significant number of new H3K27ac binding sites in anaphase/telophase, which are only present in anaphase/telophase (Fig. 1B). Combined with Hi-C data, we surprisingly found that this anaphase/telophase-specific H3K27ac binding was particularly enriched at the TAD boundaries and was even higher than interphase specific peaks (Fig. 6J), suggesting anaphase/telophase-specific function of H3K27ac at insulators. In the future, it would be interesting to determine the functional role of H3K27ac in the reformation of long-range interactions after mitosis and whether it serves to recruit architectural proteins CTCF or cohesin complex or/and provide a bookmark for domain boundaries or A-A and B-B compartments. Taken together, the increase of H3K27ac in anaphase/telophase seems to influence promoters-driven transcriptional reactivation and TAD reformation.

BRD4 is a histone acetyltransferase (HAT) that acetylates histones H3 and H4 such as H3K14ac, H3K27ac, H3K122ac, and H4K16ac (Filippakopoulos et al. 2012; Devaiah et al. 2016). It binds AEs and controls the activation of cell type-specific genes in various cell systems (Lee et al. 2017; Najafova et al. 2017; Wu et al. 2018). Prior studies have shown that BRD4 is partially retained on mitotic chromatin and its overexpression accelerates the transcriptional activation in early G1 (Dey et al. 2009; Zhao et al. 2011), suggesting its function as a transcriptional bookmark on mitotic chromatin. Recently, a genome-wide approach reveals that BRD4 widely occupies mitotic chromatin at H3K27ac sites. However, neither transcription activation nor H3K27ac mitotic binding were perturbed by mitotic-specific inhibition of BRD4 following JQ1 treatment (Behera et al. 2019). This suggests that BRD4 is dispensable for either transcriptional reactivation or H3K27ac retention during mitosis, but BRD4 may still be involved in some other regulatory function. Another recent study shows that BRD4 is indispensable for osteoblast-lineage specification during differentiation. ChIP-seq reveals a high co-occupancy of BRD4 with other TFs such as C/EBPb, TEAD1, FOSL2, and JUND at putative osteoblast-specific enhancers in human fetal osteoblasts cells, hFOB (Najafova et al. 2017). We observed that a subset of BRD4 and c-Jun associated with mitotic chromatin using immunofluorescence analysis in U2OS cells (data not shown). Although the colocalization of two TFs and genome-wide spatial information needs to be explored, these observations suggest that mitotic BRD4 may recruit TFs at cell type-specific enhancers and play a role in epigenetic memory of cell type-specific transcriptional program.

Recently, a genome-wide RNA pulse-labeling study showed that some genes are actively transcribed in prometaphase arrested cells, and described waves of gene reactivation during mitotic exit (Palozola et al. 2017). Using the same approach, we were able to observe mitotically expressed genes, as well as the hierarchy of gene activation from prometaphase to G1 phase (Fig. 4). However, we also made a set of different observations. First, while our study reveals that the largest number of genes are first activated at 60 min (cytokinesis/early G1), the other study shows it at 80 min (metaphase-anaphase), which is earlier in the cell cycle than our observation. This conflicting result can be explained by the use of different synchronization protocols. Although both studies use thymidine-nocodazole block to arrest prometaphase cells, the efficiency of cell cycle synchronization can be affected by incubation time and concentration of chemicals. Long incubation or high concentration of chemicals can prevent the ability of cells exiting mitosis and reentering the next G1 phase, and therefore can delay cells released from mitotic arrest and lead to a wide range of heterogeneous cell populations (Zieve et al. 1980). Thus, we used less nocodazole for shorter incubation time and enabled to efficiently enrich homogenous cell populations at each time point. Second, our analysis reveals that the largest number of cell type-specific genes are expressed at 60 min (cytokinesis/early G1) in a similar trend with general EU-labeled gene expression, yet the other study shows that most are expressed at 300 min (early G1), which is later than when the largest number of EU-labeled genes are first activated at 80 min. This difference may simply result from low coverage of cell type-specific genes: 113 bone-specific genes in our study and 149 liver-specific genes in their study. However, both studies are in agreement regarding the timing of activation of cell type-specific genes at early G1. Activation of cell type-specific gene expression requires appropriate cell type-specific TFs, which are mostly recruited by cytokinesis, and enhancer-mediated loops, which start to be formed after cytokinesis. Overall, in spite of a few different observations, we were able to find genes actively transcribed throughout mitosis to G1.

Multiple studies show that CTCF remains associated with mitotic chromosomes using imaging and Western blot analyses (Burke et al. 2005; Liu et al. 2017), which provide suggestive mitotic bookmarking function at insulator elements, given its essential role in TAD formation. Our ChIP-seq analysis, however, shows that CTCF binding sites are almost entirely lost during prometaphase. Dissociation of CTCF from mitotic chromatin was also observed using imaging and chromatin fractionation approaches. Phosphorylation of CTCF may explain this dissociation. In vitro assay shows that CTCF becomes highly phosphorylated during mitosis and this phosphorylation of CTCF impairs its DNA-binding activity (Sekiya et al. 2017). A recent study using a different genomic technique, CUT&RUN, which can be performed on unfixed cells, unlike ChIP-seq, reveals the loss of CTCF binding in prometaphase as well (Oomen et al. 2019). Thus, it seems unlikely that our observation is due to technical artifacts such as formaldehyde-induced fixation. Interestingly, the majority of loss of CTCF binding during prometaphase is regained by anaphase/telophase, earlier than TAD formation, and CTCF relocalizes at the TAD boundaries. Similarly, cohesin almost completely dissociates during prometaphase and rapidly reassociates as cells exit mitosis (Cai et al. 2018; Abramo et al. 2019). The loop extrusion model underlies TAD formation through cohesin-mediated loop extrusion, which is stalled at TAD boundaries due to CTCF binding (Sanborn et al. 2015; Fudenberg et al. 2016). Thus, loss of TADs during prometaphase seems to be due to loss of CTCF and cohesin and TAD reformation seems to be mediated by CTCF and cohesin recruitment at the boundaries during anaphase/telophase.

## Materials and methods

### Cell culture and cell cycle synchronization

U2OS cells were cultured in DMEM (Gibco) and 10% FBS. RPE1 cells were cultured in DMEM-F12 (Gibco) and 10% FBS. Cells were incubated at 37°C and 5% CO_2_. Cells were synchronized using thymidine and nocodazole treatments. The mitotic cells were collected by mitotic shakeoff. A-485 (10 μM; Tocris) was used to inhibit H3K27ac levels. See the Supplemental Material for detailed description.

### Immunofluorescence

Cells were grown on glass coverslips and fixed with 4% paraformaldehyde in PBS for 5 min at room temperature. Fixed cells were permeabilized with cold PBS containing 0.5% Triton X-100 for 5 min. Cells were blocked with 1% BSA in PBS for 1 h at room temperature and incubated in primary antibody in blocking buffer overnight at 4°C, followed by secondary antibody for 1 h at room temperature. Cells were then briefly stained with 1 μg/mL Hoechst 33342 (Molecular Probes H-1399) in PBS and mounted in VectaShield (Vector Laboratories). Images were acquired in a Zeiss LSM710 confocal microscope. Images were analyzed and prepared for presentation in Photoshop.

### Isolation of nuclear and cytoplasmic fractions

Cells were resuspended in hypotonic buffer (5 mM Pipes at pH 8, 85 mM KCl, 0.5% NP-40, protease inhibitor) for 10 min on ice , followed by centrifugation at 500*g* for 10 min at 4°C. The supernatant containing the cytoplasmic fraction was transferred and saved. The pellet was resuspended in lysis buffer (50 mM Hepes at pH 7.9, 5 mM MgCl_2_, 0.2% Triton X-100, 20% glycerol, 300 mM NaCl, protease inhibitor) for 30 min on ice, followed by centrifugation at 12,000*g* for 20 min at 4°C. The supernatant was collected as the nuclear fraction.

### Histone isolation and Western blotting

For histone extraction, the protocol is adapted from previous work (Shechter et al. 2007). Briefly, following nuclei isolation, soluble histones were extracted with 0.2 M HCl, followed by TCA/acetone precipitation. For Western blot analysis, protein samples were resolved in SDS-PAGE gels and transferred to PVDF membranes (Millipore). Membranes were blocked with 5% milk in TBST (0.25% Tween 20, 20 mM Tris at pH 8.0, 137 mM NaCl) and incubated with primary antibody overnight at 4°C. After three 5-min washes in TBST, HRP-conjugated secondary antibody was added for 1 h at room temperature. Membranes were visualized by SuperSignal West Pico or Femto (Thermo Fisher Scientific) reagent.

### Antibodies

The following antibodies were purchased from the indicated commercial sources: anti-H3K4me3 (Abcam ab8580), anti-H3K4me1 (Abcam ab8895), anti-H3K27ac (Abcam ab4729), anti-CTCF (Active Motif 61311), anti-spike-in (Active Motif 61686), anti-α-Tubulin (Sigma T5168), anti-Histone H3 (Cell Signaling 4499), and anti-H3S10p (Cell Signaling 3377).

### ChIP-seq

Cells (5 × 10^6^) were cross-linked with 1% formaldehyde for 10 min, and ChIP-seq was performed as described previously (Toyama et al. 2019). Spike-in was carried out according to vendor protocols (Active Motif). Briefly, 50 ng of spike-in chromatin (Active Motif 53083) was added to 25 μg of U2OS or RPE1 chromatin to incubate with 2 μg of spike-in antibody together with 5 μg of anti-H3K4me3, anti-H3K4me1, anti-H3K27ac, or anti-CTCF antibodies. DNA libraries were generated using the Kapa Hyper preparation kit for Illumina platforms (Kapa Biosystems). Libraries were sequenced in a NextSeq 500 system (Illumina). See the Supplemental Material for detailed information about data analysis, including alignment, normalization, peak calling, *cis*-REs identification, motif analysis, and Pearson correlation coefficient test.

### Spike-in control sequences and EU-RNA-seq

Biotinylated spike-in controls and samples for EU-RNA-seq were prepared as previously described (Palozola et al. 2017). Briefly, U2OS cells were pulse-labeled with 0.5 mM EU for 35 min at 37°C. Total RNA was harvested using Trizol (Ambion) and purified using miRNeasy (Qiagen). Click reaction was performed to conjugate biotin to the EU-labeled RNA using Click-iT nascent RNA capture kit (Invitrogen). Two biotinylated spike-in control RNAs were added to 1.5 μg of each biotinylated sample (0.36 ng of control #1 and 0.036 ng of control #2). Biotin-EU-RNAs, including spike-in controls, were pulled down with streptavidin-coated magnetic beads. For validation of biotin-RNA spike-in controls, cDNA was generated using the SuperScript VILO cDNA synthesis kit (Invitrogen) followed by qPCR. For sequencing, cDNA libraries were generated using the Ovation human FFPE RNA-seq multiplex system. Multiplexed pair-end sequencing was performed on a NextSeq 500 instrument (Illumina). See the Supplemental Material for detailed information about data analysis, including alignment, normalization, identification of hierarchy of gene expression, and GO enrichment analysis.

### Hi-C

Hi-C was performed using the in situ method as previously described (Rao et al. 2014). Briefly, U2OS cells (2 × 10^6^) were cross-linked with formaldehyde. Chromatin was digested with a restriction enzyme MboI (NEB), biotinylated with biotin-ATP (Life Technologies), and then ligated with T4 DNA ligase (NEB). DNA was purified and sheared with Covaris LE220 instrument (Covaris). Biotinylated DNA interactions were pulled down with Dynabeads MyOne Streptavin T1 Beads (Life Technologies) and sequenced in a NovaSeq 6000 sequencing system (Illumina). See the Supplemental Material for detailed information about data analysis.

### Accession numbers

The Gene Expression Omnibus accession number for the ChIP-seq, EU-RNA-seq, and Hi-C data reported in this study is GSE141139.

## Supplementary Material

Supplemental Material
