# Supplementary material for: Dynamic regulation of histone modifications and long-range chromosomal interactions during postmitotic transcriptional reactivation
Source: Genes Dev. 2020 Jul 1;34(13-14):913–30. doi: 10.1101/gad.335794.119 (PMC7328517; doi:10.1101/gad.335794.119)
Supplement: Supplemental Material [file supp_34_13-14_913__index.html]

Dynamic regulation of histone modifications and long-range chromosomal interactions during postmitotic transcriptional reactivation — Supplemental Material 

# Dynamic regulation of histone modifications and long-range chromosomal interactions during postmitotic transcriptional reactivation

## Supplemental Material

- Supplemetal\_Data.pdf
- Supplemental\_Table\_S1.xlsx
- Supplemental\_Table\_S2.xlsx
- Supplemental\_Table\_S3.xlsx
