# Supplementary material for: Dynamic regulation of histone modifications and long-range chromosomal interactions during postmitotic transcriptional reactivation
Source: Genes Dev. 2020 Jul 1;34(13-14):913–30. doi: 10.1101/gad.335794.119 (PMC7328517; doi:10.1101/gad.335794.119)
Supplement: Supplemental Material [file supp_gad.335794.119_Supplemental_Data_.pdf]

Supplemental Material

Supplemental Materials and Methods

Supplemental References

Supplemental Figures S1-S7

Supplemental Materials and Methods

Cell culture and cell cycle synchronization

U2OS osteosarcoma cells were cultured in DMEM (Gibco) and 10% FBS. To obtain mitotic cells, U2OS cells were treated with filter-sterilized 2.5 mM thymidine (Sigma) for 30 h, washed, and released into thymidine-free fresh medium. After 10 h release from S phase arrest, the cells were incubated with 20 ng/ml nocodazole (Sigma) for 4 h. To enrich for mitotic cells, rounded floating cells were collected with shake-off. To release the cells from arrest, mitotic cells were washed three times with PBS to remove nocodazole and then incubated in fresh medium in suspension culture.
RPE1 hTERT immortalized cells were cultured in DMEM-F12 (Gibco) and 10% FBS. RPE1 cells were synchronized in S phase by double thymidine block with 2 mM thymidine for 14 h, release into fresh media for 10 h, and 2 mM thymidine for 14 h. After 6 h release from thymidine block, cells were synchronized in prometaphase using 20 ng/ml nocodazole for 4 h cells. The mitotic cells were then collected by mitotic shake-off. Anaphase/telophase enriched cells were collected after 35 min incubation in the nocodazole-free medium.

ChIP-seq Analysis

Raw read quality was checked using FastQC and contamination was checked with FastqScreen and BLAST. Reads were mapped to an hg19 (human) and dm6 (fly spike-in) combined genome using the STAR aligner version 2.5.3b (Dobin et al. 2013). Mapping was carried out using default parameters (up to 10 mismatches per read, and up to 9 multi-mapping locations per read). The genome index was constructed using the gene annotation supplied with the hg19 and dm6 Illumina iGenomes (iGenomes online. Illumina. 2015. http://support.illumina.com/sequencing/sequencing_software/igenome.html) collection and sjdbOverhang value of 100. After mapping, fly reads were quantified separately and used to determined spike-in normalization factors for all samples assuming a constant amount of spike-in added. Final normalization factors(f) were calculated by renormalizing each cell cycle phase(p) to the interphase(I) within each replicate(r) for each antibody(a):

$$f_{p,r,a}=(|human_{p,r,a}|+|fly_{p,r,a}|)|fly_{p=I,r,a}|/|fly_{p,r,a}|(|human_{p=I,r,a}|+|fly_{p=I,r,a}|)$$

Peaks were identified using HOMER (Heinz et al., 2010), using default parameters and input condition as background reference. Normalized read counts for peaks were calculated using the fragments per kilobase per million mapped reads (FPKM) normalization multiplied by the spike-in factors. Low coverage peaks were filtered out (average log2 normalized read counts < 3 across all phases for H3K27ac, H3K4me3, and H3K4me1, and average log2 normalized read counts < 1 across all phases for CTCF, due to lower quality ChIP for CTCF). Then peaks were defined as present in a

phase if the average normalized expression in that phase was at least the ¾ the maximum normalized read count across all 3 phases. Peak overlap across cell cycle phases was calculated using HOMER mergePeaks routines with a maximum distance for merging of 1000 bp.

Genomic Element Analysis

Phase-specific genomic elements were defined according to the presence of H3K27ac, H3K4me1, and H3K4me3 normalized peaks in each phase and their proximity to the transcription start sites (TSS) of annotated RefSeq genes. Promoters (Pr) were defined as any regions containing H3K4me3 within 1kb of the TSS of known genes. Primed enhancers (PE) were defined as regions containing only H3K4me1 peaks at a distance of more than 1kb away from the TSS of known genes. Active enhancers (AE) were defined as regions containing both H3K4me1 and H3K27ac peaks at a distance of more than 1kb away from the TSS of known genes. HOMER mergePeaks was used to find all combinations of overlaps between peaks in each phase and HOMER annotatePeaks was used to determine the distance to the nearest TSS.

To calculate the distribution of normalized read coverage around the center of genomic elements (Pr, AE, PE), HOMER annotatePeaks was used with a window size of +/- 3kb and a bin size of 100 bp. Spike-in normalization factors were applied and the average read coverage of replicates was shown as a histogram.

The set of promoters and enhancers maintained during prometaphase and those lost in prometaphase, then regained in anaphase/telophase, was determined with HOMER

mergePeaks applied to the elements directly. The known motif enrichment analysis was carried out using HOMER findMotifsGenome.pl with -size given.

EU-RNA Analysis

Raw read quality was checked using FastQC and contamination was checked with FastqScreen and BLAST. Reads were mapped to an hg19 (human) using the STAR aligner version 2.5.3b (Dobin et al. 2013). FPKM (fragments per kilobase per million mapped reads) gene expression was quantified across the entire gene with HOMER analyzeRepeats. In addition, reads mapping to spike-in sequences were used to calculate the normalization slope based on the expected concentrations of the spike-ins as was done previously (Palozola et al. 2017). Expression values were further log2 transformed, averaged across replicates, and NoEU control expression values were subtracted from EU values at each time point. Next, time point-specific genes were identified as genes whose expression exceeds 50% of the average expression across all times (excluding asynchronous) and continues to exceed 50% for the duration of the time course. Asynchronous genes were defined as those that were not time point specific and whose values were at least twice as high in the asynchronous time point compared to any of the other time points. Heatmaps were generated using R libraries gplots (heatmap.2 function), or plot.matrix (plot function) with scaling across conditions. An absolute log2fold change of 1 was used to determine genes up or down with A-485. To correlate EU expression with the ChIP-seq results, we used the merged peaks from the replicates of each ChIP antibody and cell-cycle phase to quantify the EU read counts at each time point averaged across both replicates and normalized for peak size.

These values were then correlated to the average ChIP read counts for that antibody and cell-cycle phase normalized to the peak size. Gene Ontology functional enrichment analysis was performed using DAVID (Dennis et al. 2003).

Hi-C data analysis

Sequencing reads generated from samples of eight time points with two replicates were subjected to alignment and processing as previously described (Dixon et al. 2018), and contact matrices were constructed and normalized by matrix balancing. We have performed standard identification of features in Hi-C datasets, including compartments (Lieberman-Aiden et al. 2009), TADs (Dixon et al. 2012), loops (Rao et al. 2014) and insulation scores (Crane et al. 2015). Annotated TADs of 360 minutes samples were normalized by size and divided into 20 bins for ChIP-seq peak mapping for all time points. Local minimums were identified for insulation vectors of all samples for plotting.

Supplemental References

Crane E, Bian Q, McCord RP, Lajoie BR, Wheeler BS, Ralston EJ, Uzawa S, Dekker J, Meyer BJ. 2015. Condensin-driven remodelling of X chromosome topology during dosage compensation. *Nature* **523**: 240-244.

Dennis G, Sherman BT, Hosack DA, Yang J, Gao W, Lane HC, Lempicki RA. 2003. DAVID: Database for Annotation, Visualization, and Integrated Discovery. *Genome Biol* **4**: P3.

Dixon JR, Selvaraj S, Yue F, Kim A, Li Y, Shen Y, Hu M, Liu JS, Ren B. 2012. Topological domains in mammalian genomes identified by analysis of chromatin interactions. *Nature* **485**: 376-380.

Dixon JR, Xu J, Dileep V, Zhan Y, Song F, Le VT, Yardımcı GG, Chakraborty A, Bann DV, Wang Y et al. 2018. Integrative detection and analysis of structural variation in cancer genomes. *Nat Genet* **50**: 1388-1398.

Dobin A, Davis CA, Schlesinger F, Drenkow J, Zaleski C, Jha S, Batut P, Chaisson M, Gingeras TR. 2013. STAR: ultrafast universal RNA-seq aligner. *Bioinformatics* **29**: 15-21.

Heinz S, Benner C, Spann N, Bertolino E, Lin YC, Laslo P, Cheng JX, Murre C, Singh H, Glass CK. 2010. Simple combinations of lineage-determining transcription factors prime cis-regulatory elements required for macrophage and B cell identities. *Mol Cell* **38**: 576-589.

Lieberman-Aiden E, van Berkum NL, Williams L, Imakaev M, Ragoczy T, Telling A, Amit I, Lajoie BR, Sabo PJ, Dorschner MO et al. 2009. Comprehensive mapping of long-range interactions reveals folding principles of the human genome. *Science* **326**: 289-293.

Palozola KC, Donahue G, Liu H, Grant GR, Becker JS, Cote A, Yu H, Raj A, Zaret KS. 2017. Mitotic transcription and waves of gene reactivation during mitotic exit. *Science* **358**: 119-122.

Rao SS, Huntley MH, Durand NC, Stamenova EK, Bochkov ID, Robinson JT, Sanborn AL, Machol I, Omer AD, Lander ES et al. 2014. A 3D map of the human genome

at kilobase resolution reveals principles of chromatin looping. *Cell* **159**: 1665-1680.

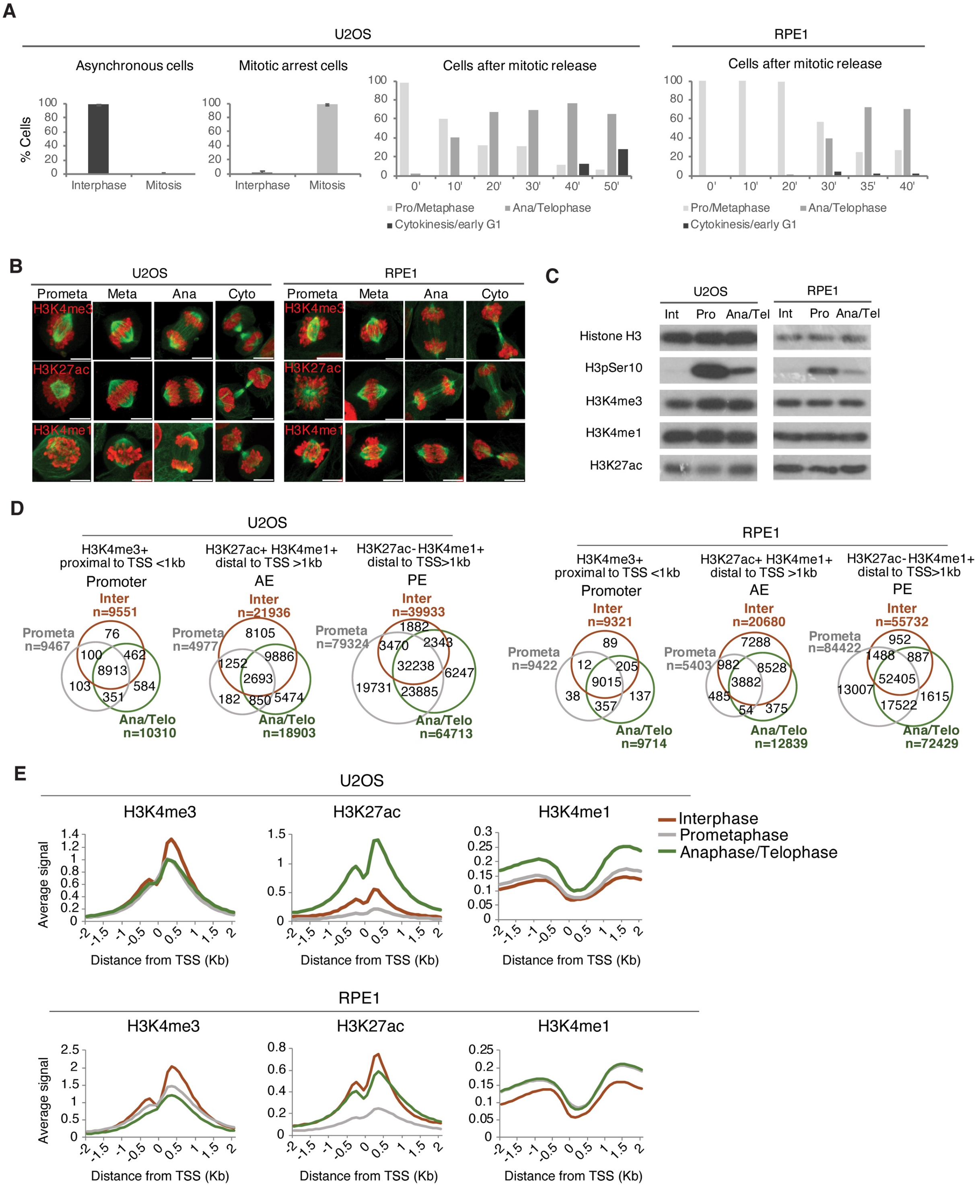
A
U2OS
RPE1
Asynchronous cells
Mitotic arrest cells
Cells after mitotic release
% Cells
Interphase
Mitosis
Pro/Metaphase
Ana/Telophase
Cytokinesis/early G1
B
Prometa
Meta
Ana
Cyto
H3K4me3
H3K27ac
H3K4me1
C
Int
Pro
Ana/Tel
Histone H3
H3pSer10
D
H3K4me3+ proximal to TSS <1kb
Promoter
H3K27ac+ H3K4me1+ distal to TSS >1kb
AE
H3K27ac- H3K4me1+ distal to TSS>1kb
PE
Inter
n=9551
Prometa
n=9467
Ana/Telo
n=10310
n=21936
n=4977
n=18903
n=39933
n=79324
n=64713
n=9321
n=9422
n=9714
n=20680
n=5403
n=12839
n=55732
n=84422
n=72429
E
Average signal
Distance from TSS (Kb)
Interphase
Prometaphase
Anaphase/Telophase

Figure S1. Retention of histone modifications on mitotic chromatin. (A) Synchronization was monitored by microscopy using both Hoechst and α-Tubulin staining. The number of U2OS cells was counted in the left panel: mitotic contamination in asynchronous cells (left), mitotic index in mitotic arrest cells (middle), and mitotic release from prometaphase for indicated time (right). The number of cells released from mitotic arrest was also counted in RPE1 cells (right panel). (B) Immunofluorescence images show the presence of histone modifications on mitotic chromatin. U2OS and RPE1 were stained for α-Tubulin (green) and either H3K4me3, H3K27ac, or H3K4me1 (red). Images were taken at mitotic cells naturally occurred. Bars, 10 μm. (C) Western blot analysis shows an abundance of H3K4me3, H3K4me1, and H3K27ac in histone extracts from interphase (Int), prometaphase (Pro), and anaphase/telophase (Ana/Tel) enriched cells. Histone H3 was used as the loading control. H3pser10 was used as the mitotic marker. (D) Venn diagram showing the number of promoters, AEs, and PEs in each cell cycle phase. Regulatory elements were defined as indicated in U2OS (left panel) and RPE1 (right panel) cells. (E) Histogram showing ChIP-seq reads of H3K4me3, H3K27ac, and H3K4me1 relative to TSS during interphase (orange), prometaphase (gray), and anaphase/telophase (green) in U2OS (upper panel) and RPE1 (bottom panel) cells.

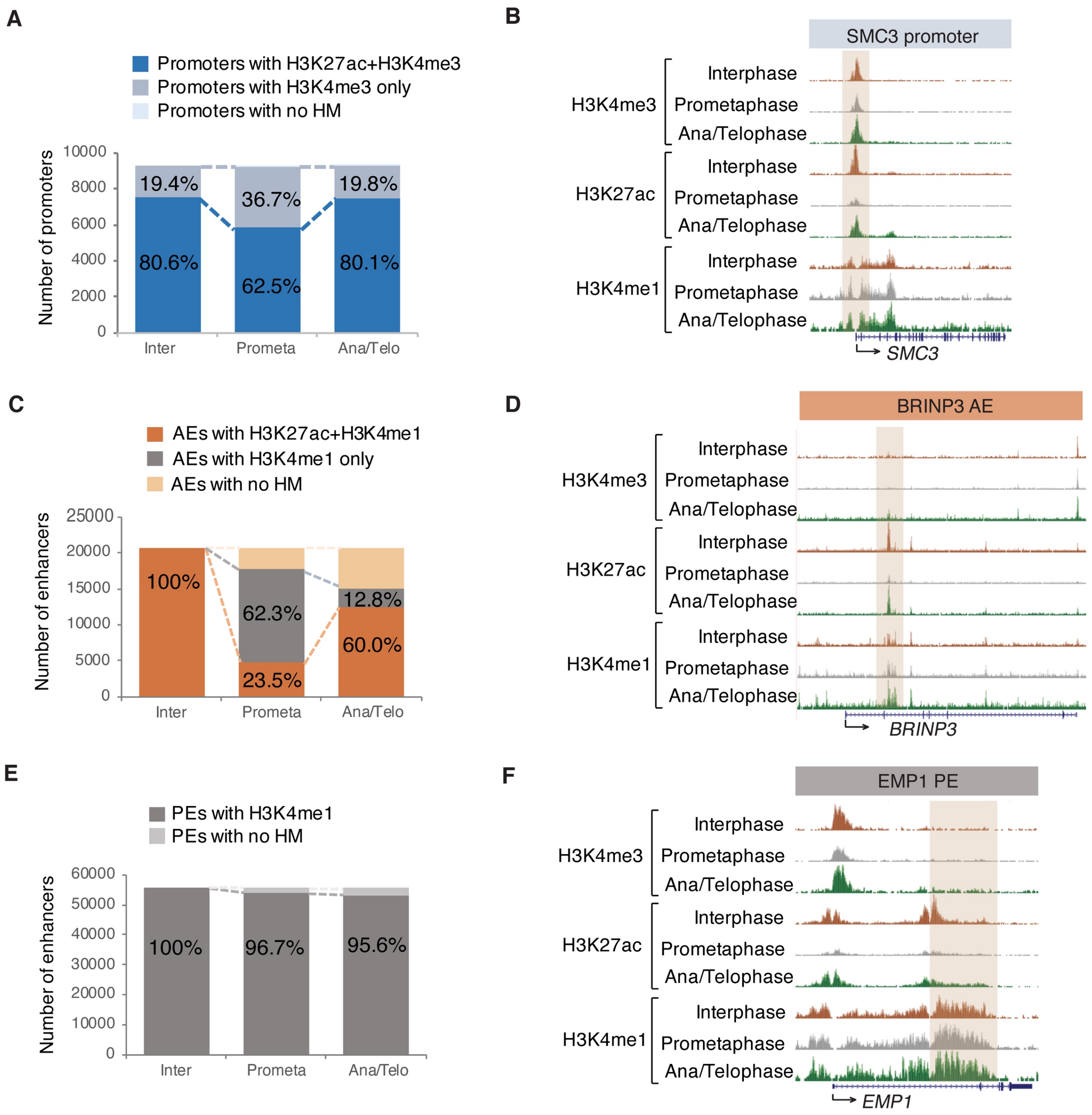

Figure S2. Histone methylations bookmark promoters and enhancers in the absence of H3K27ac during prometaphase in RPE1 cells. (A) Quantification of interphase promoters that are also detected in prometaphase or in anaphase/telophase. Promoters harboring both H3K27ac and H3K4me3 are shown in blue and H3K4me3 only in light blue. Promoters not containing any histone modification are shown in the lightest blue

color. (B) ChIP-seq tracks at the promoter of the *SMC3* gene for H3K4me3, H3K27ac, and H3K4me1 during interphase, prometaphase, and anaphase/telophase. (C) Quantification of interphase AEs that are also detected in prometaphase or in anaphase/telophase. AEs harboring both H3K27ac and H3K4me1 are shown in orange and H3K4me1 only in gray. AEs not containing any histone modification are shown in light orange. (D) ChIP-seq tracks at AE of *BRINP3* gene for H3K4me3, H3K27ac, and H3K4me1 during interphase, prometaphase, and anaphase/telophase. (E) Quantification of interphase PEs that are also detected in prometaphase or in anaphase/telophase. PEs harboring H3K4me1 are shown in gray. PEs not containing any histone modification are shown in light gray. (F) ChIP-seq tracks at PE of *EMP1* gene for H3K4me3, H3K27ac, and H3K4me1 during interphase, prometaphase, and anaphase/telophase. Peaks are highlighted by brown boxes. Abbreviations: interphase (Inter), prometaphase (Prometa), anaphase/telophase (Ana/telo), histone modification (HM).

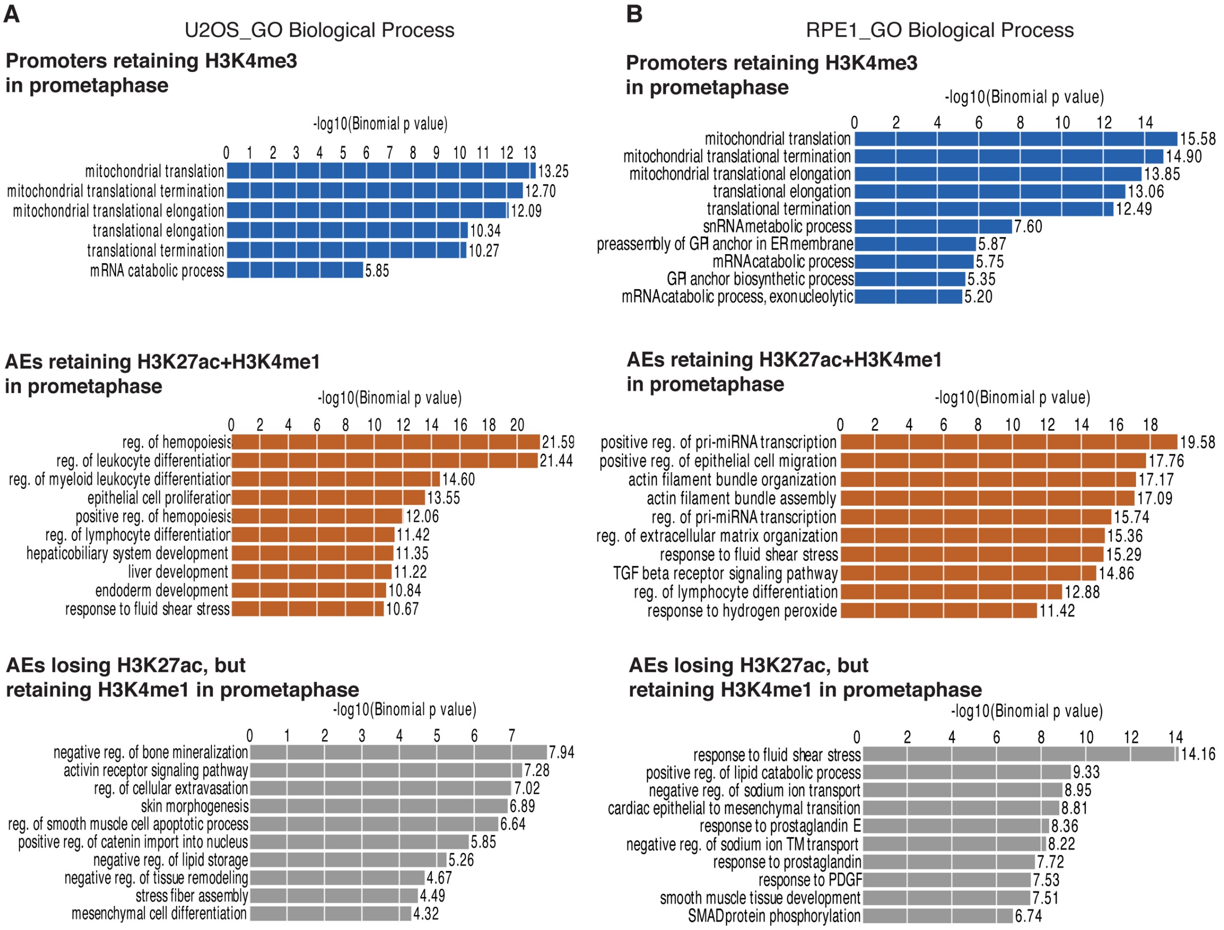

Figure S3. H3K4me1 remains at enhancers of cell type-specific genes during prometaphase in U2OS and RPE1. (A, B) Top 10 Gene Ontology (GO) annotations enriched in promoters retaining H3K4me3 (blue), AEs retaining both H3K27ac and H3K4me1 (orange) and AEs losing H3K27ac, but retaining H3K4me1 (gray) during prometaphase in U2OS (A, left panel) and RPE1 (B, right panel).

Kang_FigS4

A

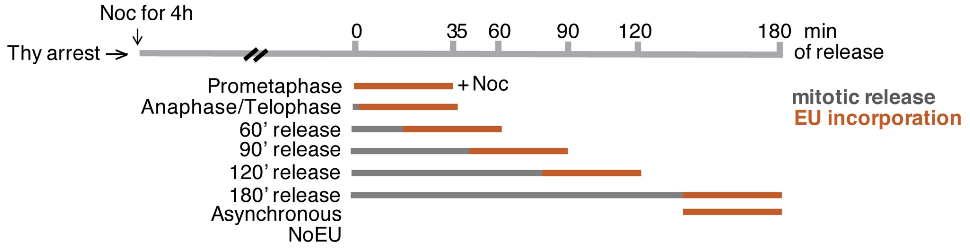

B

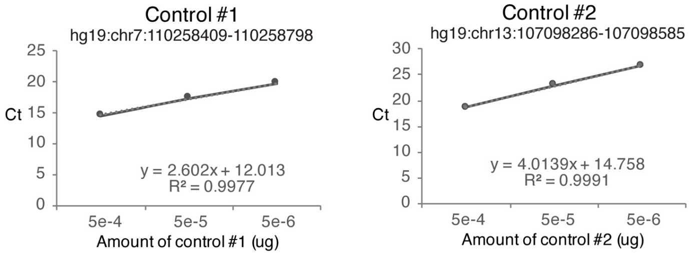

C

			0 min	35 min	60 min	90 min	120 min	180 min
Pearson correlation R between ChIP-seq signals at promoter and gene expression at each time point	H3K4me3	Inter	0.37	0.25	0.49	0.46	0.46	0.46
		Prometa	0.36	0.24	0.48	0.45	0.45	0.45
		Ana/Telo	0.41	0.28	0.52	0.50	0.51	0.50
	H3K27ac	Inter	0.29	0.16	0.35	0.35	0.36	0.38
		Prometa	0.44	0.30	0.53	0.54	0.55	0.53
		Ana/Telo	0.47	0.32	0.60	0.58	0.58	0.56
	H3K4me1	Inter	-0.20	-0.15	-0.27	-0.24	-0.25	-0.25
		Prometa	-0.18	-0.11	-0.24	-0.21	-0.21	-0.19
		Ana/Telo	-0.15	-0.09	-0.20	-0.17	-0.17	-0.15

Pearson correlation: 0.60, 0.50, 0.40, 0.30, 0.20, 0.10

Figure S4. EU-RNA-seq reveals nascent RNA during mitosis-G1. (A) Schematic of EU-RNA-seq strategy illustrating pulse-labeling during mitosis-G1. (B) Validation of spike-in controls by RT-qPCR. The indicated amount of control #1 or control #2 was added at 1.5 ug of asynchronous biotin-EU-RNA and pulled down with streptavidin-coated magnetic beads. Ct values of control #1 or control #2 show the control sequences are pulled down proportionally on streptavidin-coated magnetic beads in the presence of total asynchronous RNA. (C) Pearson correlation coefficient (R) was determined between the transcriptional level at each time point and the ChIP-seq reads of H3K4me3, H3K27ac, and H3K4me1 in interphase (Inter), prometaphase (Prometa), and

anaphase/telophase (Ana/Telo) at the promoter. Positive correlations are shown in red color (R > 0).

Kang_ FigS5

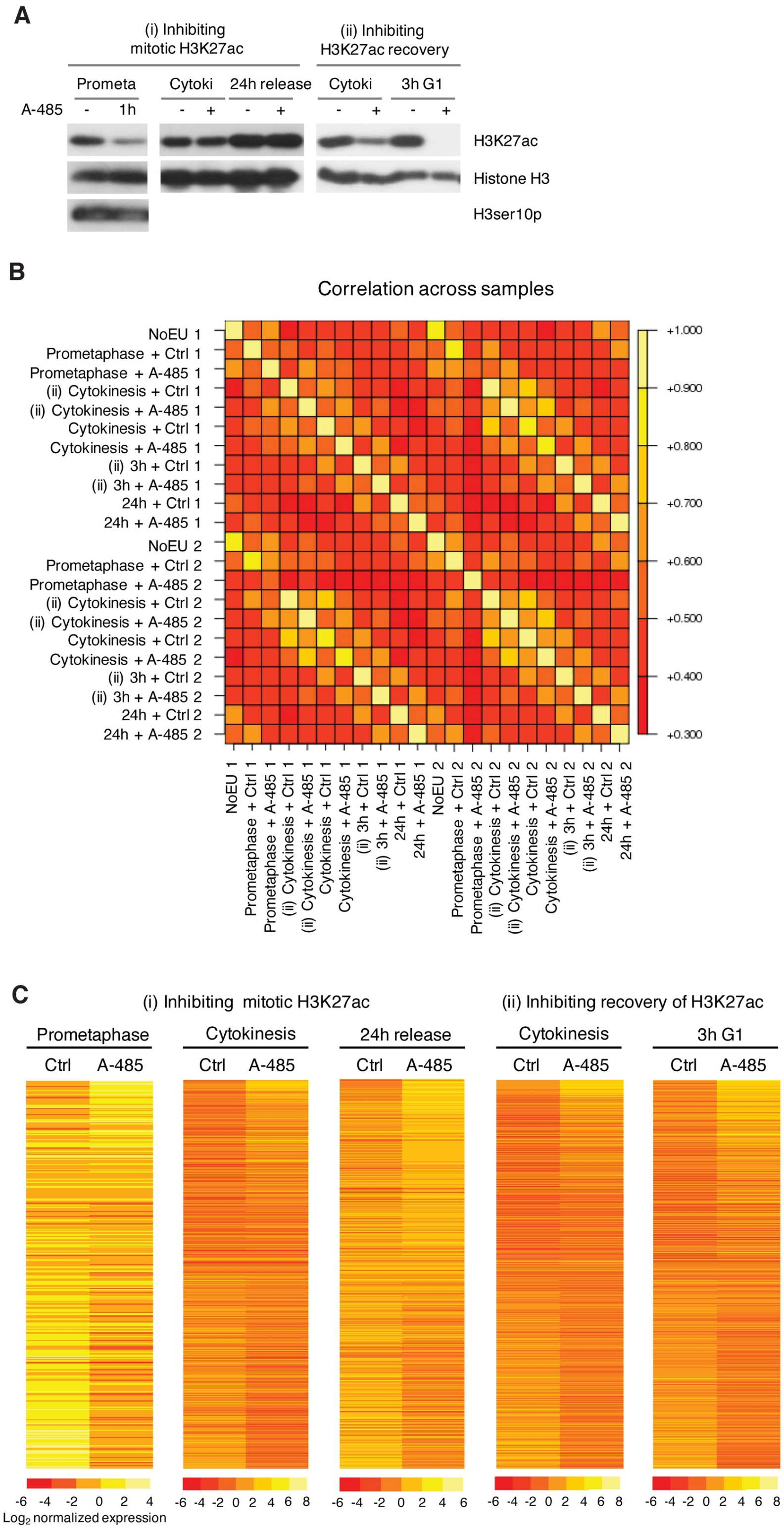
A
(i) Inhibiting mitotic H3K27ac
(ii) Inhibiting H3K27ac recovery
Prometa
Cytoki
24h release
Cytoki
3h G1
A-485
- 1h
- +
- +
- +
- +
H3K27ac
Histone H3
H3ser10p
B
Correlation across samples
NoEU 1
Prometaphase + Ctrl 1
Prometaphase + A-485 1
(ii) Cytokinesis + Ctrl 1
(ii) Cytokinesis + A-485 1
Cytokinesis + Ctrl 1
Cytokinesis + A-485 1
(ii) 3h + Ctrl 1
(ii) 3h + A-485 1
24h + Ctrl 1
24h + A-485 1
NoEU 2
Prometaphase + Ctrl 2
Prometaphase + A-485 2
(ii) Cytokinesis + Ctrl 2
(ii) Cytokinesis + A-485 2
Cytokinesis + Ctrl 2
Cytokinesis + A-485 2
(ii) 3h + Ctrl 2
(ii) 3h + A-485 2
24h + Ctrl 2
24h + A-485 2
+1.000
+0.900
+0.800
+0.700
+0.600
+0.500
+0.400
+0.300
C
(i) Inhibiting mitotic H3K27ac
(ii) Inhibiting recovery of H3K27ac
Prometaphase
Cytokinesis
24h release
Cytokinesis
3h G1
Ctrl A-485
Ctrl A-485
Ctrl A-485
Ctrl A-485
Ctrl A-485
-6 -4 -2 0 2 4
Log2 normalized expression
-6 -4 -2 0 2 4 6 8
-6 -4 -2 0 2 4 6
-6 -4 -2 0 2 4 6 8
-6 -4 -2 0 2 4 6 8

Figure S5. Inhibition of mitotic H3K27ac and H3K27ac recovery by A-485 alters gene expression pattern. (A) Western blot analysis shows A-485 treatment during mitosis inhibits H3K27ac levels in histone extracts from prometaphase (Prometa) (left panel). H3K27ac levels in cytokinesis (Cytoki) and 24h release cells after wash out A-485 from mitotic arrest (middle panel). A-485 treatment upon releasing cells from mitotic arrest inhibits H3K27ac levels in cytokinesis (Cytoki) and 3h G1 cells (right panel). U2OS cells were treated with 1h of 10μM A-485 or EtOH as a control. Histone H3 was used as the loading control. (B) Heatmaps of Pearson correlations between replicates of the same conditions. (C) Heatmap demonstrating the differential gene expression pattern between control (EtOH) and A-485 treatment in each cell cycle phase.

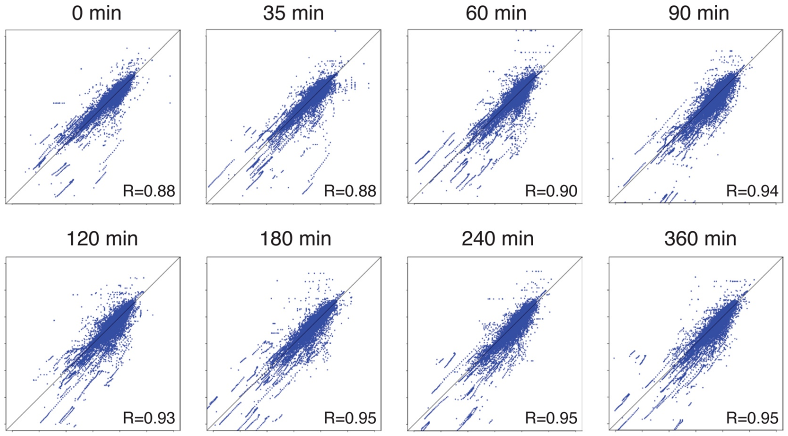

Figure S6. Hi-C analysis during mitosis-G1. Pearson correlation of compartment eigen vector between two replicates at each time point and 1 Mb bin size.

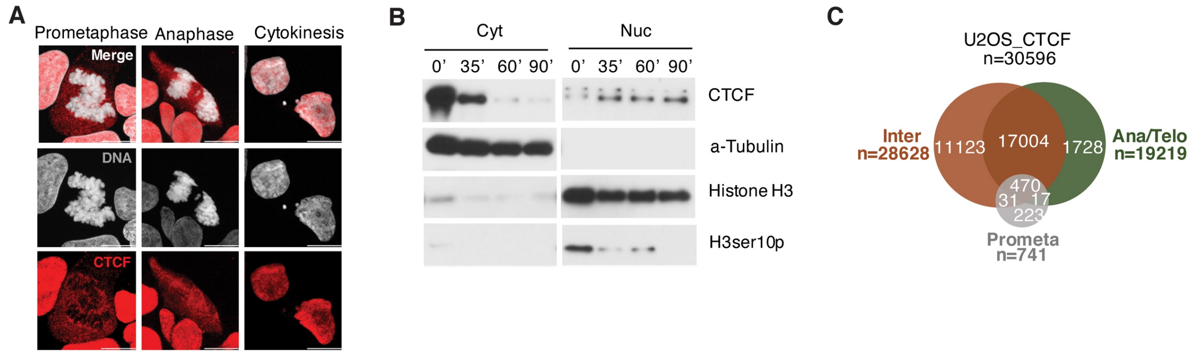

Figure S7. Global level change of CTCF during mitosis. (A) Immunofluorescence analysis of CTCF in mitotic phases. U2OS cells were stained for Hoechst (gray) and CTCF (red). CTCF was largely dispersed to the cytoplasm during prometaphase. (B) Western blot showing CTCF levels in nuclear or cytoplasmic fractions isolated from U2OS cell lysates after thymidine-nocodazole mitotic arrest or nocodazole washout. α-Tubulin and Histone H3 were used as the loading control in the cytoplasmic or nuclear fraction, respectively. H3ser10p was used as the mitotic marker. (C) Venn diagram showing the number of CTCF binding sites in interphase (Inter), prometaphase (Prometa), and anaphase/telophase (Ana/Telo) in U2OS cells, with n representing the number of observed CTCF peaks in each cell cycle phase.
